# Genomics of *Tenacibaculum* Species in British Columbia, Canada

**DOI:** 10.3390/pathogens12010101

**Published:** 2023-01-06

**Authors:** Joseph P. Nowlan, Ashton N. Sies, Scott R. Britney, Andrew D. S. Cameron, Ahmed Siah, John S. Lumsden, Spencer Russell

**Affiliations:** 1 Center for Innovation in Fish Health, Vancouver Island University, Nanaimo, BC V9R 5S5, Canada; 2 Department of Pathobiology, University of Guelph, Guelph, ON N1G 2W1, Canada; 3 Department of Biology, University of Regina, Regina, SK S4S 0A2, Canada; 4 Institute for Microbial Systems and Society, Faculty of Science, University of Regina, Regina, SK S4S 0A2, Canada; 5 BC Center for Aquatic Health Sciences, Campbell River, BC V9W 2C2, Canada

**Keywords:** phylogenetics, *de novo* assembly, virulence, antimicrobial resistance, diversity, *Tenacibaculum*, mouthrot, Atlantic salmon

## Abstract

*Tenacibaculum* is a genus of Gram-negative filamentous bacteria with a cosmopolitan distribution. The research describing *Tenacibaculum* genomes stems primarily from Norway and Chile due to their impacts on salmon aquaculture. Canadian salmon aquaculture also experiences mortality events related to the presence of *Tenacibaculum* spp., yet no Canadian *Tenacibaculum* genomes are publicly available. Ribosomal DNA sequencing of 16S and four species-specific 16S quantitative-PCR assays were used to select isolates cultured from Atlantic salmon with mouthrot in British Columbia (BC), Canada. Ten isolates representing four known and two unknown species of *Tenacibaculum* were selected for shotgun whole genome sequencing using the Oxford Nanopore’s MinION platform. The genome assemblies achieved closed circular chromosomes for seven isolates and long contigs for the remaining three isolates. Average nucleotide identity analysis identified *T. ovolyticum*, *T. maritimum*, *T. dicentrarchi*, two genomovars of *T. finnmarkense*, and two proposed novel species *T. pacificus* sp. nov. type strain 18-2881-A^T^ and *T. retecalamus* sp. nov. type strain 18-3228-7B^T^. Annotation in most of the isolates predicted putative virulence and antimicrobial resistance genes, most-notably toxins (i.e., hemolysins), type-IX secretion systems, and oxytetracycline resistance. Comparative analysis with the *T. maritimum* type-strain predicted additional toxins and numerous C-terminal secretion proteins, including an M12B family metalloprotease in the *T. maritimum* isolates from BC. The genomic prediction of virulence-associated genes provides important targets for studies of mouthrot disease, and the annotation of the antimicrobial resistance genes provides targets for surveillance and diagnosis in veterinary medicine.

## 1. Introduction

*Tenacibaculum* is a genus of Gram-negative bacteria that are ubiquitous in marine environments and have beneficial, neutral, or negative interactions with marine organisms [1]. Experimental exposures and circumstantial evidence indicates multiple species of *Tenacibaculum* as the causative agents of disease in fishes of economic or cultural significance (e.g., salmonids [2,3,4], temperate basses [5], and flatfishes [6]). Fishes affected by ‘tenacibaculosis’ often display epidermal lesions that can be accompanied by the development of yellow plaques and abnormal behaviours [1]. The signs and severity of tenacibaculosis appear to depend on the species or strain of *Tenacibaculum* [2,3,4]; the host species, health-status, and life-stage; and the environmental conditions. In British Columbia (BC), Canada, a regional presentation of tenacibaculosis called ‘mouthrot’ presents as oral plaques and ulcerations on Atlantic salmon (*Salmo salar* L.). Mouthrot is treated based on the presence of mouthrot-associated mortality, thus preventing an improved understanding of the etiological agents at the species level. Collected isolates from outbreaks are often identified post-mouthrot treatment using PCR with bidirectional Sanger-sequencing and qPCR. Identifying genes common to pathogenic isolates will inform the mechanistic understanding of the pathogenesis of mouthrot, improving the specificity of future treatments, such as vaccines, and will enhance the design of assays for future genetic-based diagnostics.

Several *Tenacibaculum* genome assemblies are available in the literature, including species believed to be putative pathogens (e.g., *T. maritimum* NCIMB 2154^T^ [7]; *T. ovolyticum* da5A-8 and To7-Br [8,9]; *T. dicentrarchi* and *T. finnmarkense* isolates [10]; and *T. piscium* isolates [11,12]). Genomic assemblies of *Tenacibaculum* bacteria from disease outbreaks improve species identification in the context of molecular diagnostics [7,8,9,10,11,12], in contrast to traditional, cheaper, and faster techniques, such as 16S rDNA sequencing or single gene PCR, which could result in ambiguous species level predictions [1]; however, genomic techniques are expensive and require significant time to complete. With an increasing number of *Tenacibaculum* genomes world-wide, further investigations can be conducted to select targeted genes related to antimicrobial resistance and the pathogenesis of *Tenacibaculum* species. Putative virulence and antimicrobial resistance genes identified in other *Tenacibaculum* isolates [7,8,9,12] have not been investigated in BC isolates of *Tenacibaculum*, and understanding which genes are involved in disease or resistance is critical for interpreting the pathogenesis of the disease and informing treatment. Specifically, iron-binding and -uptake proteins can be key determinants of virulence in bacterial pathogens because they enable the scavenging of essential iron from the host [13]. Similarly, identifying toxin secretion systems can predict a pathogen’s potential to damage host cellular structures and functions [14]. Comparative genomics can identify putative virulence factor genes in *Tenacibaculum* spp., which can be targeted for genetic modification or deletion in *Tenacibaculum* isolates for in-vivo or cell line investigation.

Few *Tenacibaculum* genomes are completely assembled. Of the 254 *Tenacibaculum* spp. genome assemblies on the National Center for Biotechnology Information (NCBI, https://www.ncbi.nlm.nih.gov/, accessed on 28 November 2022), only nine are complete assemblies of singular, circular chromosomes (28 November 2022). This is because the short-read next-generation sequencing technologies used to sequence most *Tenacibaculum* genomes precludes the de novo assembly of the complete genomes [8,9,10,11,12,15]. Long-read sequencing platforms can overcome this limitation [15,16]; using PacBio long-read and hybrid sequencing, at least seven *Tenacibaculum* genomes have been completed (Table 1). No complete assemblies are available for *Tenacibaculum* bacteria isolated from BC waters, a globally important region for marine aquaculture. Therefore, the objective was to isolate, sequence, identify, and annotate the *Tenacibaculum* species from BC Atlantic salmon.

In this study, Oxford Nanopore Technologies (ONT) MinION long-read sequencing was applied to generate complete genomes for phylogenetically diverse *Tenacibaculum* isolates from coastal BC Atlantic salmon with mouthrot. ONT has proven increasingly successful for the assembly of complete genomes, comparative genome analysis, and the identification of antimicrobial resistance and pathogenicity determinants [17,18,19]. With robust genome assemblies, comparative phylogenomics occurred with traditional genetic loci (16S rDNA, *fusA*, *atpA*, multi-locus sequence analysis) and average nucleotide identity (ANI) to determine the species-level identity. Complete genomes also enabled the annotation of the antimicrobial resistance (AMR) and virulence genes, providing candidate genes for future virulence diagnostics and vaccine production.

**Table 1:** National Center for Biotechnology Information (NCBI, https://www.ncbi.nlm.nih.gov/, accessed on 28 November 2022) complete and incomplete *Tenacibaculum* assemblies.

Assembly Level	Sequencing Platform	Isolate	Contigs	N50 (Mb)	L50	Genbank Accession Number	Citation
Complete	PacBio Sequel	*Tenacibaculum* sp. AHE14PA	1	2.8	1	GCA_019278465.1	[20]
		*Tenacibaculum* sp. AHE15PA	1	2.8	1	GCA_019278445.1	
		*T. mesophilum* bac2	1	3.4	1	GCA_024181065.1	NA
	PacBio single-molecule real-time	*T. dicentrarchi* AY7486TD	1	2.9	1	GCA_001483385.1	[21]
	PacBio RSII	*T. todarodis strain LPB0136* ^T^	1	3.0	1	GCA_CP018155.1	[22]
	PacBio RSII and Illumina HiSeq	*T. maritimum* NCIMB 2154^T^	1	3.4	1	GCA_900119795.1	[7]
	PacBio RSII and Illumina MiSeq	*T. mesophilum* DSM 13764^T^	1	3.5	1	GCA_009362255.1	[23]
	PacBio	*T. maritimum* TM-KORJJ	1	3.3	1	GCA_004803875.1	NA
	NA	*T. jejuense strain* KCTC 22618^T^	1	4.6	1	GCA_900198195.1	NA
Incomplete	Various	*Tenacibaculum* spp.*	1 – 1196	3.9 × ^−03^–4.5	1-191	Various	NA

* Uses 245 *Tenacibaculum* species assemblies.

## 2. Materials and Methods 

### 2.1. Isolate Selection

The *Tenacibaculum* isolates were collected from ulcers identified on the jaws, flanks, or gills of Atlantic salmon during mouthrot outbreaks between 2017 and 2020. All of the isolates were cultured on *Flexibacter maritimus* media (FMM) supplemented with 50 µg × mL^−1^ of kanamycin (FMM+K). Ten isolates were classified to a species or genus-level identity based on its morphological characteristics (i.e., yellow, Gram-negative, elongated rod-shaped to filamentous); 16S rDNA sequencing using universal primers (27F (5′-AGAGTTTGATCATGGCTCAG -3′), 1492R (5′- GGTTACCTTGTTACGACTT -3′)), and species-specific qPCR tests were used for *T. dicentrarchi* and *T. finnmarkense* [24], *T. maritimum* [25], and *T. ovolyticum* [26]. All of the qPCR primers, probes, and protocols are described in previous work [24,25,26]. The PCR products using universal primers were generated using the AllTaq Master Mix Kit (QIAGEN, Hilden, Germany), according to manufacturer’s guidelines, using 100 ng of isolate DNA; the thermal profile consisted of 95 °C (5 min); 40 cycles of 95 °C (1 min), 60 °C (1 min), and 72 °C (3 min); and 72 °C (5 min). The products were cleaned (MinElute ^®^ Reaction Cleanup Kit, QIAGEN, Hilden, Germany) and underwent bidirectional Sanger sequencing (University of Alberta, Molecular Biology Facility, Edmonton, Canada). The sequences were aligned in MEGAX, and the consensus sequence was used for NCBI BLAST comparisons.

### 2.2. DNA Extractions

The selected isolates were grown in a FMM+K broth to an absorbance (A_600_) of 0.49 ± 0.11, followed by DNA extractions (gBAC Mini DNA Bacteria Kit, IBI Scientific, Iowa, USA), according to the manufacturer’s instructions. Concentrations of the extracted products were quantified using spectrophotometry (NanoVue, GE Healthcare, Illinois, USA), where A_260/280_, and A_260/230_ were also recorded. Concentrations of the extracted samples were also quantified using fluorescence (Qubit^TM^ dsDNA BR Assay kit, Invitrogen, Massachusetts, USA), according to the manufacturer’s instructions, in a BioSpectrometer^®^ fluorescence (Eppendorf, Hamburg, Germany).

### 2.3. MinION Sequencing

The isolates were subjected to Oxford Nanopore long-read MinION sequencing at the BC Center of Aquatic Health Sciences (CAHS) in Campbell River (BC, Canada). The samples underwent DNA repair and end-prep, native barcode ligation, adapter ligation and clean-up, priming and loading the flow cell using Oxford Nanopore protocol with the Native Barcoding Expansion 1–12 kit (EXP-NBD104, Oxford, England) and Ligation Sequencing kit (SQK LSK 109, Oxford, England). During the adaptor ligation step, the long fragment buffer was utilized instead of the short fragment buffer. The pooled reactions from the adapter ligation and clean-up were loaded into MinION flow-cell (FLO MIN 106D v.R9, Oxford, England) according to the Oxford Nanopore protocol.

### 2.4. MinION Post-Processing and Quality Control

FAST5 files collected from the MinION flow-cell output were basecalled using the ONT Guppy software (v.6.0.1 + 652ffd1) and the super high accuracy model (guppy/6.0.1/data/dna_r9.4.1_450bps_sup model). The subsequent outputs were demultiplexed (i.e., debarcoded) using the ONT Guppy software (v.6.0.1 + 652ffd1), according to the basic command for ‘guppy_barcoder –trim_barcodes’, except that mid-read adaptors and extra basepairs from the barcodes were trimmed using the command ‘-trim_barcodes --min_score_barcode_front 70 -q 0 --detect_mid_strand_adapter --min_score_adapter_mid 65 --num_extra_bases_trim 80′. Modified demultiplexing occurred to remove potential chimeric reads, but also to reduce nucleotide basecalled noise, which were compared and observed using NanoQC. Reads shorter than 500 bp were omitted using filtlong (v 0.2.1). The basecalled and demultiplexed barcodes were used in Nanoplot (v.1.39) to infer the basic sequencing statistics.

### 2.5. Genome Assembly and Polishing

Each isolate was assembled using Trycycler v.0.5.1 (https://github.com/rrwick/Trycycler, accessed on 28 November 2022, [27]) because Trycycler uses multiple genome assembly tools to create a consensus genomic sequence. Trycycler consists of eight steps (seven using the Trycycler pipeline and a single step using Medaka for sequence polishing (v.1.4.3, © Oxford Nanopore Technologies Ltd., Oxford, UK, 2018)):

1. Subsample each barcode independently into 12 maximally independent read sets.
2. Create an assembly with each read subset for each barcode using Flye (v.2.9, [28]), Raven (v.1.6.1, [29]), miniasm (v.0.3-r179, [30]), and minimap2 (v.2.23-r1111, [30]).
3. Cluster the contigs produced by each assembly per barcode (resulting in a hierarchical cluster dendrogram, from which subjective decisions were made).
4. Reconcile (circularize and align to a consistent start position) all of the contigs included in the cluster from the previous step per barcode.
5. Compute the multiple sequence alignments between all of the reconciled contigs per cluster.
6. Partition the initial read files (complete sets per barcode) into their appropriate clusters (i.e., chromosome reads to chromosome cluster, plasmid reads to plasmid cluster, etc.).
7. Compute the consensus for each cluster, for each barcode. Use the reconciled contigs (step 4), alignments (step 5), and raw reads for the given cluster (step 6).
8. Medaka polishing (v.1.4.3) per barcode.

Following ‘step 2′ in the Trycycler pipeline, barcodes 2, 9, and 10 were in numerous contigs that differed between the read subsets, preventing effective clustering in ‘step 3′. As a result, a single-assembler approach was used to assemble these barcodes. Two programs (Flye v.2.9, Raven v.1.6.1) were initially used, and that which produced the most contiguous assemblies was used. Post-assembly and polishing, Ori-Finder 2022 (http://tubic.tju.edu.cn/Ori-Finder2022/public/index.php/index, accessed on 4 November 2022) was used to find the predicted origins of the circular chromosomes [31].

### 2.6. Genomic Annotation

The assembled genomes for barcodes 1–10 were annotated using Bakta (v.1.4.0, https://github.com/oschwengers/bakta, [32]). A BLAST search [33] was used to identify the target genes when the gene could not be identified using Bakta. Visuals of draft annotated genomes were developed using Proksee (https://proksee.ca/, accessed on 28 November 2022), which implements CGView [34].

### 2.7. Phylogenomic Investigations

#### 2.7.1. 16S rDNA

Each annotated nucleotide FASTA file of the final assembly was mined through text-searches for 16S ribosomal DNA nucleotide sequences, where alignments on MEGAX (https://www.megasoftware.net/, accessed on 28 November 2022, [35]) occurred using MUSCLE with the ‘Toggle Conserved Sites’ set to 100% within each barcode to manually interpret the number of single nucleotide polymorphisms (SNPs) and potential insertion-deletion events (INDELs). Each unique 16S rDNA sequence underwent a nucleotide BLAST comparison on the NCBI website (https://blast.ncbi.nlm.nih.gov/Blast.cgi, accessed on 28 November 2022), with the search term ‘Organism’ limited to ‘bacteria (taxid:2)’. An alignment was generated for each unique 16S rDNA sequence using MUSCLE in MEGAX. The NCBI 16S sequences were included from type strains of *Tenacibaculum* species, *Tenacibaculum piscium* RT-G19 (OL304282.1), *Kordia algicida* OT-1^T^ (AB681152), and *Flavobacterium johnsoniae* UW101^T^ (CP000685.1). A Maximum-Likelihood phylogeny was achieved using the W-IQ-TREE Web Service (http://www.iqtree.org/, accessed on 28 November 2022) provided by the Center for Integrative Bioinformatics (Vienna, Austria). All of the phylogenies used the ‘Auto’ substitution model, which selected the model that provided the largest Bayesian Information Criteria (BIC) score, followed by 10000 ultrafast bootstrap alignments and 10000 SH-aLRT branch tests. The 16S rDNA and all of the subsequent phylogenies were visualized on ITOL (v6, https://itol.embl.de/, accessed on 28 November 2022, [36]).

#### 2.7.2. atpA and fusA

Similar to 16S rDNA, each nucleotide FASTA file was mined by text-searching for *atpA* and *fusA* genes, except that alignment to interpret single nucleotide polymorphisms were not applied as there was generally one copy for each isolate. Each gene was based on sequences described for *atpA* [37] and *fusA* [38]. Due to the limited sequence availability for *fusA*, select sequences on NCBI that identified as either gene for *Tenacibaculum* species were used in the alignments. For the generated phylogenies, *Kordia algicida strain* OT-1^T^ (NZ DS544873.1) was used instead of *Kordia algicida strain* OT-1^T^ (AB681152) as both genes were not described in this sequence. Phylogenetic comparisons were then conducted in a similar manner to the ‘16S rDNA’ methodology.

#### 2.7.3. Multilocus Sequence Analysis (MLSA)

MLSA was accomplished for each annotated barcode by text-searching for *atpA*, *dnaK*, *glyA*, *gyrB*, *infB*, *rlmN*, and *tgt*. Each sequence was then aligned by gene in MEGAX using MUSCLE and the aligned sequences were trimmed [37]. Post-trimming, the isolated sequences for each barcode were then joined in the order described by the *Tenacibaculum* PUBMLST database (https://pubmlst.org/organisms/tenacibaculum-spp (accessed on 8 November 2022)). The *Tenacibaculum* type strain MLSA profiles on the PUBMLST database were exported and included in the alignment. The NCBI sequences for each gene from the *Kordia algicida strain* OT-1^T^ (NZ DS544873.1) and the *Flavobacterium johnsoniae* strain UW101^T^ (CP000685.1) were collected, and the MLSA profiles were made in a similar manner and included in the alignment. Phylogenetic comparisons were then conducted in a similar manner to the 16S rDNA methodology.

#### 2.7.4. Average Nucleotide Identity (ANI)

The complete genomes of each barcode were compared to each other, as well as other NCBI genomes of *Tenacibaculum*, using FASTANI (v.1.33, https://github.com/ParBLiSS/FastANI, accessed on 28 November 2022, [39]). For this comparison, putative plasmid sequences were also included due to their genomic similarities to the contigs in the published assemblies [40]. The included NCBI *Tenacibaculum* genomes are presented in Table 2.

### 2.8. Virulence, Antimicrobial Resistance, and Genomic Island Investigations

Potential virulence factors were inferred using: FeGenie (v1.0, https://github.com/Arkadiy-Garber/FeGenie, accessed on 28 November 2022, [41]); and Virulence Finder v2.0 (https://cge.cbs.dtu.dk/services/VirulenceFinder/, accessed on 28 November 2022, [42,43]); where *Listeria*, *S. aureus*, *E. coli*, and *Enterococcus* were designated as ‘Select species’, the ‘Select threshold for %ID’ was 90% and the ‘Select minimum length’ was 60%. A BLAST search also occurred for the putative virulence factors described as toxins in *T. maritimum* NCIMB 2154^T^ [7] for barcodes 1–10. Subsequently, a text-search occurred in the Bakta annotated files for the toxin categories described in *T. maritimum* NCIMB 2154^T^ [7].

The potential antimicrobial resistance genes were inferred using three tools. The Comprehensive Antibiotic Resistance Database (CARD)-Resistance Gene Identifier (https://card.mcmaster.ca/analyze/rgi, accessed on 28 November 2022, [44]), using ‘perfect, strict, and loose hits’ and ‘include nudge’; ResFinder 4.1 (https://cge.food.dtu.dk/services/ResFinder/, accessed on 28 November 2022, [33,45,46]) using ‘Other’ for the selected species, and ‘Assembled Genome/Contigs’ for the type of reads; and ARG-ANNOT v6 using the default settings [47].

Two tools were used to identify the potential genomic islands (GI) for each barcode: IslandViewer4 (https://www.pathogenomics.sfu.ca/islandviewer, accessed on 28 November 2022, [48]) and GYPSy (v1.1.3, [49]). IslandViewer4 predicts the GI using genomic signature deviation (GC content (IslandPath-DIMOB) and codon usage (SIGI-HMM)); the presence of transposases, integrases, and flanking tRNA (IslandPath-DIMOB); and comparative genomics (Islandpick). IslandViewer4 also requires a reference genome when the submitted assembly is in more than one contig. For IslandViewer4, barcodes 2, 9 and 10 were mapped to *Tenacibaculum dicentrarchi* AY7486TD (now *T. finnmarkense*) or *Tenacibaculum maritimum* NCIMB 2154^T^ prior to analysis. GYPSy predicts the GI using genomic signature deviation (GC content and codon usage (Colombo-SIGI-HMM)); the presence of transposase genes; flanking tRNA (HMMR3); factors for virulence, metabolism, AMR, and symbiosis; and comparative genomics. For GYPSy, a reference genome is required for the comparative genomics. Barcode 1 was compared against *Tenacibaculum ovolyticum* DSM 18103^T^; barcodes 2, 3, 4, 5, 6, 7, and 8 were compared against *Tenacibaculum finnmarkense* AY7486TD; and barcodes 9 and 10 were compared against *Tenacibaculum maritimum* NCIMB 2154^T^. Putative plasmids were excluded for GI comparisons.

### 2.9. Gene Content Investigation

To investigate the content of each barcode and to identify the sets of genes shared between barcodes, the annotated files for each genome were compared using Panaroo (v1.3.0, https://github.com/gtonkinhill/panaroo, accessed on 28 November 2022, [50]) using the default settings (i.e., 95% similarity threshold) and a reduced similarity threshold (i.e., 80%).

## 3. Results

### 3.1. Isolate Selection

Based on the aforementioned criteria, one *T. ovolyticum*, two *T. maritimum*, two *T. dicentrarchi*, three *T. finnmarkense*, and two non-described *Tenacibaculum* species were selected (Table 3). All of the isolate DNA extractions used for the MinION sequencing had concentrations above 100 ng × µL^−1^ (spectrophotometry) and 20 ng × µL^−1^ (Qubit fluorometry), and absorbance ratios between 2–2.2 (A_260/A280_) and 2–2.4 (A_260/A230_).

### 3.2. MinION Post-Processing and Quality Control

All of the isolates were assigned a unique barcode for sequencing (Table 3). The sequencing statistics post-sequencing and –processing indicated a basecalling accuracy greater or equal to 96.8% (Mean Q-score ≥ 15), and over 84 x coverage of the estimated genome size (Table 4).

### 3.3. Genome Assembly and Annotation

**Figure 1:**
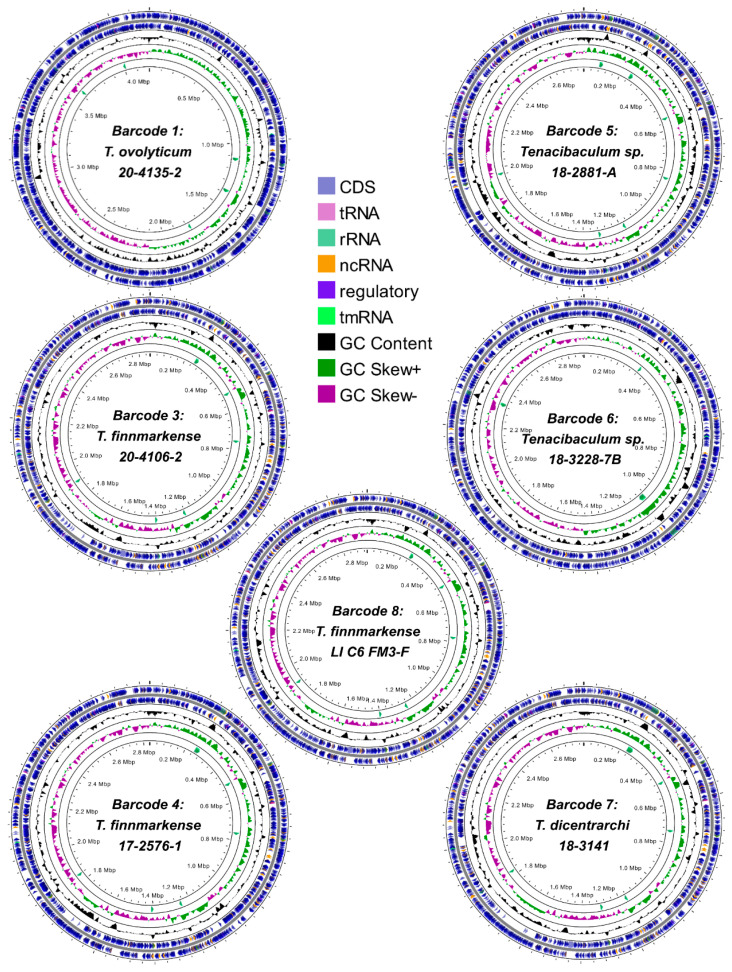
Circular *Tenacibaculum* chromosome plots assembled from Nanopore sequence data. Genomic elements are illustrated by Proksee (https://proksee.ca/, accessed on 28 November 2022). Orientation and origins are based on the *rpiB* gene. Characters displayed include coding DNA sequences (CDS), transfer-RNA (tRNA), ribosomal-RNA (rRNA), non-coding RNA (ncRNA), transfer-messenger RNA (tmRNA). Tracks outward in include characters of the genome, guanine-cytosine (GC) content, GC skews (+/−), and rRNA.

The de novo assembly of the Nanopore reads generated closed singular chromosomes for seven of the ten barcodes, with sizes ranging between 2.7–4.2 Mb. Barcodes 2 and 9 had 2–3 chromosomal contigs between 0.7–1.9 Mb (Table 5), based on alignments to the *T. dicentrarchi* AY7486TD (now *T. finnmarkense*) and *T. maritimum* NCIMB 2154^T^ circular chromosomes (Figure S1). Barcode 10 assembled into 45 contigs, preventing the confident classification of the chromosomal versus extrachromosomal contigs. Orifinder predicted origins of replication adjacent to the *rpiB* gene in six of the seven circular chromosomes, and near *rpiB* in the remaining assembly. As such, the *rpiB* locus was used to orient all of the circular chromosomes (Figure 1). Barcodes 2, 3, 4, 8, and 9 also contained smaller, circular contigs between 2–154 kb (Table 5). The genomic images from barcodes 1 and 3–8 can be found in Figure 1, with high-resolution images in Figure S2. The Bakta annotated nucleotide locus tags are provided in Table S1.

### 3.4. Phylogenetic Resolution Varies with Tenacibaculum Identification Methods

To establish the taxonomic identity of the genomic assemblies, several tools were applied (i.e., ANI, MLSA, *fusA*, *atpA*, 16S) that are used to identify *Tenacibaculum* species. The results are described in the order of highest to lowest species resolution. For downstream figures, colours denoting the species level predictions for each barcode are based on the highest resolution comparison (i.e., ANI analysis). The locations for each used genomic sequence are available in Table S2.

#### 3.4.1. Average Nucleotide Identity (ANI)

Using ANI analysis with a 95% threshold to determine the species based on the previous *Tenacibaculum* research [11,12], barcode 1 had the highest percentage of nucleotide identity with *T. ovolyticum*, barcodes 2 and 7 had the highest percentage of nucleotide identity with *T. dicentrarchi*, barcodes 3, 4, and 8 had the highest percentage of nucleotide identity with *T. finnmarkense*, and barcodes 9 and 10 had the highest percentage of nucleotide identity with *T. maritimum* (Table 6, Table S3). Barcodes 5 and 6 were unlike any of the compared assemblies and were below the 95% threshold required to determine a species-level identity (Table 6, Table S3).

#### 3.4.2. Multilocus Sequence Analysis (MLSA)

**Figure 2:**
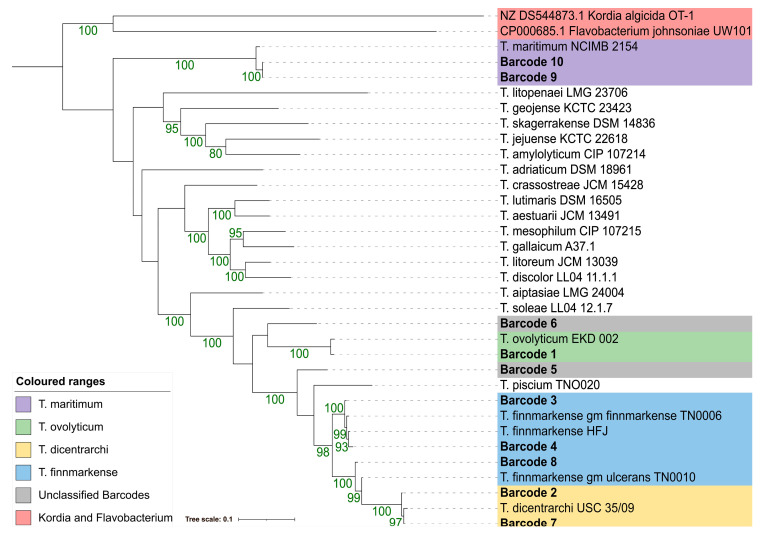
Multilocus sequence (*atpA*, *dnaK*, *glyA*, *gyrB*, *infB*, *rlmN*, and *tgt*) maximum-likelihood phylogeny of barcodes 1–10 using a GTR+F+I+G4 model. Branch lengths are proportional to the phylogenetic distance. Bootstrap values (10000 ultrafast bootstraps and 10000 SH-aLRT tests) above 80% are represented by green values at the branch. Non-barcode or outgroup sequences are derived from *Tenacibaculum* PUBMLST database (https://pubmlst.org/organisms/tenacibaculum-spp, accessed on 28 November 2022), and *Korida* and *Flavobacterium* sequences were obtained from NCBI (https://www.ncbi.nlm.nih.gov/, accessed on 28 November 2022).

Similar to ANI, an MLSA phylogeny using concatenated sequences of *atpA*, *dnaK*, *glyA*, *gyrB*, *infB*, *rlmN*, and *tgt* resulted in unambiguous species level predictions for barcodes 1–4 and 7–10 (Figure 2). These primarily mirrored the species assignments made using ANI; however, *T. finnmarkense* was grouped in a paraphyletic clade. Barcodes 5 and 6 could not be confidently classified but resolved closest to *T. piscium* and *T. ovolyticum,* respectively.

#### 3.4.3. atpA and fusA

**Figure 3:**
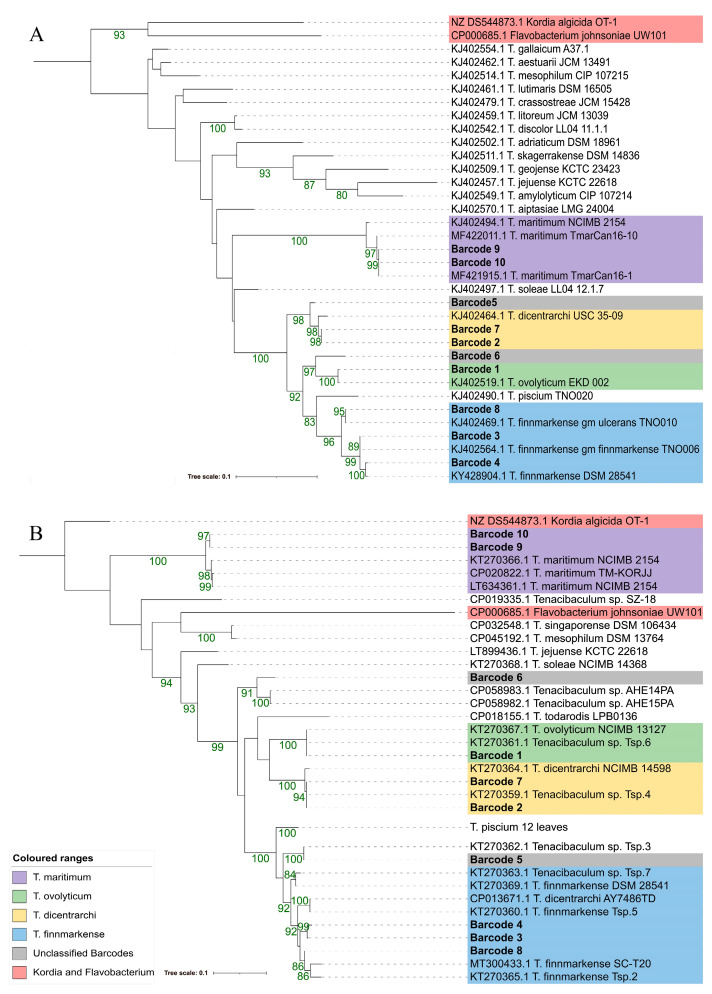
*AtpA* (**A**) and *fusA* (**B**) maximum-likelihood phylogenies of barcodes 1–10 using GTR+F+G4 and TVM+F+G4 models respectively. Branch lengths are proportional to the phylogenetic distance. Bootstrap values (10,000 ultrafast bootstraps and 10000 SH-aLRT tests) above 80% are represented by green values at the branch. Non-barcode sequences were obtained from NCBI (https://www.ncbi.nlm.nih.gov/, accessed on 28 November 2022).

*AtpA* was annotated in nine out of ten isolates and was identified using text-searches. A BLAST comparison was needed to identify the gene in barcode 4. *FusA* was annotated for eight out of ten isolates using text-searches, but a BLAST comparison was needed to identify the gene in barcode 4 and 5. Similarly to when using ANI and MLSA, the same species-level predictions could be made for barcodes 1–4 and 7–10 using both *atpA* and *fusA*. Based on the branch length, using *atpA* and a named *Tenacibaculum* species, barcodes 5 and 6 were positioned closest to *T. dicentrarchi* and *T. ovolyticum,* respectively. In contrast, when using *fusA* and a named *Tenacibaculum* species, barcodes 5 and 6 were positioned closest to *T. finnmarkense* and *T. ovolyticum*. A *Flavobacterium* sequence was clustered within the *Tenacibaculum* species for *fusA*; however, the prediction was not confident and the branch length was greater by an order of two magnitudes, relative to the sister branch (Figure 3).

#### 3.4.4. 16S rDNA

**Figure 4:**
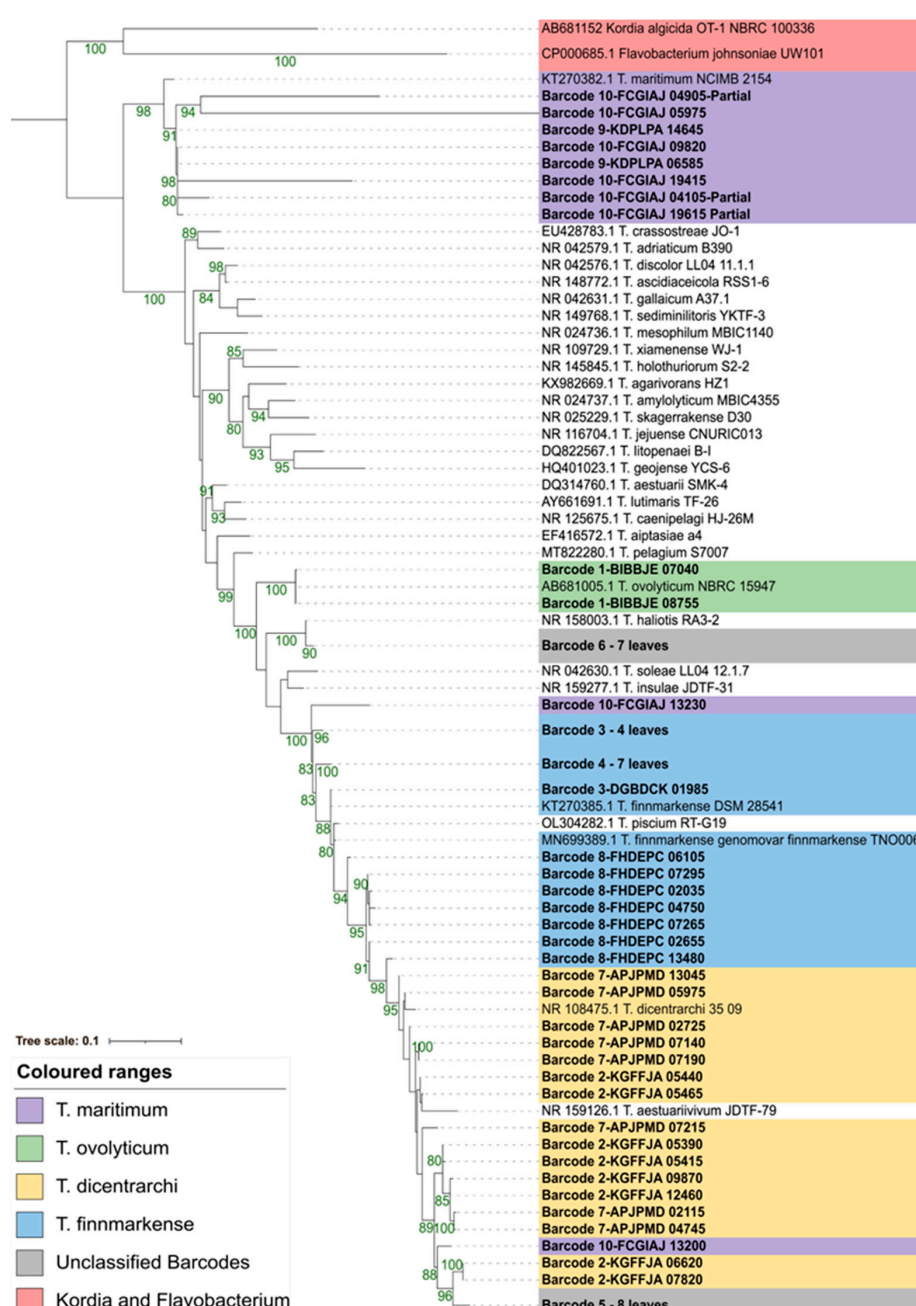
16S rDNA maximum-likelihood phylogeny of barcodes 1–10 using a TVM+F+I+G4 model. Branch lengths are proportional to phylogenetic distance. Bootstrap values (10000 ultrafast bootstraps and 10000 SH-aLRT tests) above 80% are represented by green values at the branch. Non-barcode sequences were obtained from NCBI (https://www.ncbi.nlm.nih.gov/, accessed on 28 November 2022).

Each barcode had six to nine copies of 16S rDNA (Table 7). There were also variable SNPs (≤34) and INDELs (≤14) among the 16S loci within each genome (Table 7). The multiple partial 16S sequences prevented SNP and INDEL calling for barcode 10. All of the 16S rDNA sequences most closely matched the *Tenacibaculum* species. Depending on the 16S rDNA copy used in the BLAST comparison, the closest species match could change for a given barcode (Table 6), which was also supported by the maximum-likelihood phylogenetic comparisons (Figure 4). When using both BLAST comparisons (Table 6) and 16S phylogenies (Figure 4): barcode 1 was most similar to *T. ovolyticum*; barcodes 9 and 10 were generally most similar to *T. maritimum*; however, the species level designations for barcodes 2–4 and 7–8 could not be confidently determined, but were clustered in a paraphylogeny around *T. dicentrarchi*, *T. finnmarkense*, and other *Tenacibaculum* species. Based on branch length, barcodes 5 and 6 were positioned closest to *T. dicentrarchi* and *T. haliotis*, respectively.

### 3.5. Virulence, Antimicrobial Resistance, and Genomic Islands

#### 3.5.1. Virulence Related Genes

FeGenie predicted between 40 and 77 iron-related genes per barcode that passed the bitscore cutoff and fell into one of seven categories (i.e., Iron Storage, Iron Gene Regulation, and Iron Acquisition (Siderophore synthesis, Iron Transport, Siderophore Transport, Siderophore Transport potential, and Heme Transport)) (Table S4). The Virulence Finder tool did not find any notable matches within barcodes 1–10. Each category of putative virulence factors previously described as toxins in *T. maritimum* NCIMB2154^T^ [7] were identified by a BLAST search in barcodes 9 and 10 (Table S5). A manual search among the annotated genes for the putative virulence factors described as toxins in *T. maritimum* NCIMB2154^T^ [7] identified hemolysins in all of the barcodes, barcodes 1–8 had variable matches to several categories of the described toxins, and barcodes 9 and 10 matched all of the categories (Table S5).

#### 3.5.2. Antimicrobial Resistance Genes

CARD-RGI identified between 1–4 strict matches and 142–244 loose matches for barcodes 1–10 (Table S6). The strict matches included a *tetT*, *tetQ*, or *tetB* like gene in barcodes 1–8, and a *vanT*, *vanX*, or *vanY* like gene in barcodes 1–3 and 5–10. Manual searches of the annotated genes in the barcodes indicated the presence of *tetR* and *tetQ* in barcodes 1–8 and 1–10, respectively. No ARG or resistance phenotypes were predicted using ResFinder and ARG-ANNOT.

#### 3.5.3. Genomic Islands

IslandViewer4 and GYPSy are tools that utilize codon-usage, sequence composition, mobile element presence, and genomic comparisons to infer the locations of genomic islands (GI). IslandViewer4 predicted between 3–9 GI within barcodes 1–8, and 18 and 16 GI for barcodes 9 and 10, respectively (Table S7). In contrast to IslandViewer4, GYPSy predicted between 5–26 GI within barcodes 1–10 (Table S7). GYPSy often identified more tRNA and transposases, with the exception of barcode 3. Beyond determining the GI, GYPSy also predicted that 1–10 islands in each barcode were related to pathogenicity, 0–10 islands were related to antimicrobial resistance, 0–6 islands were related to metabolism, and 0–3 islands were related to symbiosis (Table S7).

### 3.6. Gene Content Investigation

When comparing all 10 barcodes, thus considering several *Tenacibaculum* species, 191 and 973 core-genes (99–100% of barcodes) were predicted at the 95% and 80% gene cluster similarity thresholds, respectively (Table 8). Larger core-gene sets were identified in the comparisons within a single species and when comparing *T. dicentrarchi* to *T. finnmarkense* (Table 8).

## 4. Discussion

### 4.1. Genomic Assembly Provides Novel Circular Genomes

Using Oxford Nanopore long-read sequencing technology, seven of the ten *Tenacibaculum* genomes were assembled into singular, circular chromosomes. This increases the number of complete *Tenacibaculum* assemblies available for future comparisons and provides novel genomes isolated from marine waters off the coast of BC, Canada. Several studies have suggested a hybrid approach to genome construction where long-read sequencing establishes the genome structure and short-read sequencing offers high per-base accuracy [51,52,53]. For example, a hybrid approach was used on select *Tenacibaculum* isolates (*T. maritimum* NCIMB 2154^T^ (GCA_900119795.1) [7], *T. mesophilum* DSM 13764^T^ (GCA_009362255.1) [23]) and provided high-quality circular chromosomes. Future research will focus on using hybrid approaches to sequence and assemble BC *Tenacibaculum* genomes. A limitation of this study includes the fact that three chromosomes could not be completely assembled, while an unreconciled result of this study includes that the estimated number of nucleotides for barcodes 9 and 10 were ~ 4.2 Mb, which is greater than previously described (i.e., ~ 3.4 Mb) in *T. maritimum* [54,55]. This limitation and unreconciled result could be due to the quality of the template DNA, the extraction methodology, the sequencing platforms used, the genome assembly criteria, and potential contamination.

### 4.2. Variable Genomic Resolution and Novel Species

The genomic comparisons between barcodes 1–10 and the type strains of *Tenacibaculum* yielded phylogenetic insights similar to other published work. It is well established that 16S rDNA sequences can have limited use when comparing *Tenacibaculum* sequences [24,37,38,56]; however, 16S rDNA qPCR can distinguish genetically distinct species such as *T. maritimum* [25] and *T. ovolyticum* [26]. While other genes (i.e., *fusA* and *atpA*) can distinguish *Tenacibaculum* species [38,56], given the fewer sequences available in the databanks, it is unknown if these genes will experience the same limitations as 16S rRNA as more sequences are produced and deposited. For *fusA,* an outgroup was clustered within the *Tenacibaculum* genus. Increasing the number of outgroups often provides more robust phylogenies [57,58], and comparing more *Flavobacterium* and *Kordia* isolates could cluster outgroups away from *Tenacibaculum*.

The species-level identification and confidence generally improved as the amount of genome that were compared increased. ANI provided unambiguous, confident species-level predictions for all of the barcodes that belong to a known *Tenacibaculum.* The phylogenetic placement of barcodes 3 and 4 with *T. finnmarkense* gm. *finnmarkense* TNO006 and barcode 8 closer to *T. finnmarkense* gm. *ulcerans* TNO010 suggest that *atpA*, *fusA*, and MLSA may also be able to predict previously established genomovars (11); however, more work is needed to verify this inference. The ANI analysis supported the MLSA results: barcodes 3 and 4 had ~98.2% and ~96.85% nucleotide identity to *T. finnmarkense gm. finnmarkense* TNO006 and *T. finnmarkense gm. ulcerans* TNO010, while barcode 8 had ~96.8% and ~98.6% nucleotide identity to the aforementioned *T. finnmarkense* genomovars, respectively. The percent of nucleotide identities distinguishing *T. finnmarkense* genomovars were the same as Olsen et al., 2020 [11]. A limitation of using MLSA and ANI analysis for diagnostics includes the cost to complete each technique per isolate. In the field, dozens of isolates could be cultured from one collection and can cost thousands of dollars to process, depending on the number of isolates. For cost-effective diagnostic-based research, genomic investigations could establish the fewest genetic targets to reliably determine species identity, which could include sequencing *fusA* or *atpA* paired with other genes, or using species-specific genes for a multiplex PCR. Using MLSA and ANI analysis, barcode 1 was *T. ovolyticum*; barcodes 2 and 7 were *T. dicentrarchi*; barcodes 3 and 4 were *T. finnmarkense* gm. *finnmarkense*; barcode 8 was *T. finnmarkense* gm. *ulcerans*; and barcodes 5 and 6 had no similar matches but were within the genus *Tenacibaculum,* representing a novel species; and barcodes 9 and 10 were *T. maritimum*.

Barcodes 5 and 6 were unique to any of the defined and compared *Tenacibaculum* species using ANI. Both of these barcodes had less than 95% nucleotide identity to any of the compared *Tenacibaculum* species using ANI, which passes the threshold used to determine species [11,12,39], and has been used to establish novel *Tenacibaculum* species, such as *T. piscium* [11]. Previous research has used a single locus, often a 16S sequence, to identify *Tenacibaculum* species, while other biological, chemical, and biochemical traits are supplementary. As a result, it is proposed that barcode 5 (*Tenacibaculum* sp. 18-2881-A) be denoted as *Tenacibaculum pacificus* (pacificus; L. neut. adj. pacificum, peaceful; named after the Pacific Ocean (L. Mare Pacificum)) strain 18-2881-A^T^ and barcode 6 (*Tenacibaculum* sp 18-3228-7B) be denoted as *Tenacibaculum retecalamus* (*retecalamus*; L. noun rete-calamum, net-pen) strain 18-3228-7B^T^.

### 4.3. Putative Virulence Factors of BC Tenacibaculum Species

#### 4.3.1. Iron-Related Genes

Iron-related proteins are necessary for basal physiological processes in prokaryotes and eukaryotes, while virulent microbes utilize similar proteins to induce disease. FeGenie predicted numerous iron-related proteins concerning storage, gene-regulation, and acquisition. Similar predicted proteins are found in Chilean *T. dicentrarchi* [59] and *T. piscium* isolates [12], and the *T. maritimum* type strain [7], indicating that mechanisms to utilize iron between these species, and potentially among the genus, may be similar.

All ten barcodes contained several PF00210-Ferritin-like domain proteins, a non-haem iron storage protein providing vital physiological functions [60], including protection from oxidative stress by sequestering iron and limiting oxyradical formation [60,61]. A study exposing mice *to Salmonella enterica serovar* (*sv.*) *Typhimurium* demonstrated low survival rates (20% survived to d28) using the wild-type, increased survival rates (60% survived until d28) using mutants without ferritin B, and no mortality using mutants without ferritin A and B, and bacterioferritin [62].

All ten barcodes had predicted iron-regulating proteins, including proteins similar to Fur, DtxR, FecR, PchR, PvdS, and Yqil. Iron regulatory proteins have roles in essential physiological processes and in regulating virulent iron-related proteins. For example, *S. enterica sv. Typhimurium* mutants without *fur* were avirulent in mice [62] and *fur* knockouts in *Vibrio cholera* experienced reduced growth in contrast to the parental strain or ectopic complemented mutants within mice [63].

Numerous predicted proteins were related to iron acquisition; all of the barcodes had proteins relating to iron, siderophore, and heme-related transport. Most notably, all of the barcodes contain an HmuY substrate-binding protein, which is a putative heme-binding lipoprotein associated with the outer membrane [64,65], is associated with virulence [66,67], and has also been described in other *Tenacibaculum* genomes [7,12,59]. In a study, *hfpY* co-transcribed with *hfpR* produced a protein related to HmuY in *F. psychrophilum*, that contributed to host colonization and disease severity [67]. Wild-type bacteria killed all of the exposed rainbow trout, *hfpY* and *hfpR* knockout mutants killed 70% and 40% of fish, respectively, and *hfpY* and *hfpR* ectopic complemented mutants killed all the fish [67].

Iron acquisition proteins concerning siderophore synthesis were only predicted in barcodes 1 (*T. ovolyticum*), 9 and 10 (*T. maritimum*), while all barcodes encoded proteins with predicted functions for siderophore utilization. Not all bacteria produce siderophores but can often utilize them; the presence of *E. coli* enterobactin or siderphore producing *Maribacter luteus* KLE1011 bacteria influenced the presence and appearance of *M. polysiphoniae* KLE1104 [68], a bacteria which does not produce siderophores but has the ability to utilise the small molecular weight proteins and bind insoluble Fe(III). In another study, three strains of *T. maritimum* produced iron-sequestering compounds and each could use the compounds secreted by the other two [69]. Potential iron-related virulence genes in *Tenacibaculum* species warrant investigation as no genetic knockout in-vivo research has occurred to date.

#### 4.3.2. Transport and Secretion Systems

Transport systems are responsible for moving nutrients and proteins within the cell and across cell membranes, as well as moving toxins to the bacterial surface [14]. Similar to *T. maritimum* NCIMB 2154^T^ [7], the ABC-type transport, Sec-independent transport (Sec), and twin-arginine transport (Tat) proteins were present in the Bakta annotations of barcodes 1–10. Sec and Tat transport systems are universal but are often involved in virulence [14]. Type IX secretion system proteins were annotated in all ten barcodes; however, type IV and VI secretion system proteins were identified in barcodes 1, 4, 8, 9, and 10, and type II and III secretion system proteins were also present in barcode 1. Type IX secretion systems are described in other *Tenacibaculum* assemblies of *T. maritimum* NCIMB 2154^T^ [7], *T. finnmarkense* AY7486TD [10,70], *T. dicentrarchi* isolates [59], *T. piscium* isolates [12] and *T. ovolyticum* To-7Br [8,9], and therefore could be conserved within the genus. Previous *T. maritimum* NCIMB 2154^T^ exposure studies induced less than 30% mortality in Atlantic salmon under select conditions [2], but other Canadian *T. maritimum* isolates caused greater mortality, including TmarCan15-1 (100 and 75% mortality in shedders [S] and cohabitants [C]), TmarCan16-1 (100% mortality in S and C), TmarCan16-5 (>80 and 30% mortality in S and C) and *T. maritimum* 2.1C (barcode 9) (>70 and >60% mortality in S and C) [2,4]. The observed pathogenicity of *T. maritimum* 2.1C (barcode 9) could be related to the presence of type IV and VI secretion system proteins, suggesting that more work is needed to establish how virulence factors among the *Tenacibaculum* species are transported.

#### 4.3.3. Toxins

Various toxins, including a cholesterol-dependent cytolysin (i.e., hemolysin), have been described to be encoded by *T. maritimum* [7]. All ten barcodes in this study encoded a highly similar toxin (Table S5). Barcodes 9 and 10 (*T. maritimum*) encoded proteins similar to each category of toxin described [7], including: collagenase [70,71], ceramidase [72], sphingomyelinase [73], chondroitinase AC [74], streptopain family protease [70,75], sialoglycan degradation [76], and cholesterol-dependent cytolysins [70,77] (Table S5). In contrast to barcodes 1–8, barcodes 9 and 10 had several proteases with a C-terminal secretion signal. C-terminal secretions signals are often utilized in type I secretion systems (i.e., ABC-type transport systems) [14] and type IX secretion systems [78] in Gram-negative bacteria, and can also help chaperone virulence factors [79,80,81]. In this study, a putative M12B family metalloprotease that contained a C-terminal secretion signal was also described in barcodes 9 and 10. Similar M12B-family metalloproteases are a putative virulence factor in *F. psychrophilum* [82], are produced by other pathogenic microbes [83], and are in the venom of several eukaryotes [84,85,86,87,88].

### 4.4. Putative Antimicrobial Resistance Determinants of BC Tenacibaculum Species

Measuring antimicrobial resistance among isolates helps understand if trends for resistance are developing in a population. The antibiotic florfenicol is commonly used to treat mouthrot among Canadian Atlantic salmon; however, no studies have identified florfenicol resistance among Canadian *Tenacibaculum* isolates. Studies using florfenicol minimum inhibitory concentration (MIC) on 80 Canadian *T. dicentrarchi* isolates determined that all of the tested isolates were within the wild-type cut-off value (i.e., 16 µg × mL^−1^) [89]. Similar work in Chile described a *T. dicentrarchi* isolate with greater MIC for florfenicol beyond their wild-type cut-off (i.e., 4 ug × mL^−1^) [90]. The genomic investigations for barcodes 1–10 did not identify genes or proteins known to confer ‘resistance’ to florfenicol (i.e., FloR, FexA, Cfr [91]). However, the proteins described to confer resistance to oxytetracycline (i.e., TetQ [92] and TetR [93,94]) were identified. Other genomic investigations have described similar *tetQ* or *tetR* genes in *T. dicentrarchi* [59], *T. piscium* [12], and *T. aestuarii* [95]. Other proteins annotated in all ten barcodes may help confer a basal tolerance or resistance to antimicrobials, including, but not limited to: efflux pumps (multidrug resistance protein NorM, multidrug ABC transporter, CusA/CzcA family heavy metal efflux RND transporter); peroxide stress resistance (peroxide stress resistance protein YaaA, superoxide dismutase); and transcriptional regulators (multiple antibiotic resistance protein MarR). The exact mechanism by which several *Tenacibaculum* species possess a basal level of tolerance or resistance is unknown.

### 4.5. Numerous Genomic Islands among Tenacibaculum Species

Genomic islands (GI) were identified within all ten barcodes, indicating that several *Tenacibaculum* species may obtain novel genetic material through horizontal transmission. When using GYPSy, the authors advise using a genome within the same species that is also non-pathogenic [49]. However, the comparisons in this study are limited as few complete *Tenacibaculum* genomes are available and it is not well known if these genomes are pathogenic. It has been suggested to use multiple genomic island tools (i.e., GYPSy and IslandViewer4) to obtain the greatest accuracy in predicting genomic islands [91]. Both tools identified typical components of genomic islands, such as transposases and integrases; however, the tRNA associated with genomic islands was primarily identified using GYPSy, which is comparable to using both tools in *E. coli* CFT073 [96]. Few phage-related proteins were identified in the genomic islands of barcodes 1–8, but between 12 and 24 were predicted in *T. maritimum*. Phages specific to *T. maritimum* have been identified [97], and the transmission of the genes between the two should be investigated. In barcodes 2, 3, 6, and 7, a bacteriophage abortive protein (i.e., AbiH) or an abortive infection bacteriophage resistance protein was identified. The presence of an abortive protein indicates that the bacteria could have acquired methods to defend against phage-related infection, such as in *E. coli* [98] or *Lactococcus lactis* [99]. GYPSy predicted several genomic islands to be pathogenicity islands in all ten barcodes, with barcodes 1, 2, 5, 6, 7, 9, and 10 also having antimicrobial resistance islands. The occurrence of virulence- and resistance-related genes in the GIs indicates that the horizontal transmission of these genes could contribute to reoccurring infections with several species. More research is needed to investigate how these putatively horizontally acquired genes were obtained, if they can be transferred, and if they contribute to virulence, antimicrobial resistance, or tolerance.

### 4.6. Gene Content Analysis Indicating Diversity among Tenacibaculum Species

In contrast to previous studies [10,54,55], the present study compared the gene content across four *Tenacibaculum* species, where only 191 and 973 loci comprised the core genome at 95% and 80% gene cluster similarity thresholds, respectively. A small core genome has been recorded in *Lactobacillus* spp., with 266 core genes [100]. In contrast, larger core-genomes have also been recorded for *Legionella* spp., *Piscirickettsia* spp. and *Francisella* spp., with 886 and 1,732 and 692 core-genes, respectively [101]. Few highly similar genes consisting of the core-genome at 95%, but more intermediately similar genes at 80%, indicate substantial genetic diversity across the genus. In contrast to previous studies [10,54,55], the different core-genome sizes also likely occurred because the genome sizes vary across the *Tenacibaculum* species (i.e., 2.7 to 4.2 Mb).

Previous gene content analysis predicted 2013, 1947, and 1818 CDS of the core-genomes for three *T. dicentrarchi* isolates, four *T. finnmarkense* isolates, and both groups, respectively [10]. In the present study, 2140 ± 0, 2047.5 ± 6.4, 1924.5 ± 4.9 core-genes were identified within *T. dicentrarchi*, *T. finnmarkense*, and both groups, respectively, at 95% and 80%. Large core-genomes within and between the two species in both studies suggest that *T. finnmarkense* and *T. dicentrarchi* are genetically similar and may have similar interactions with the environment.

Gene content studies comparing *T. maritimum* core-genomes are consistent in the literature. In one study, 2116 core-genes accounting for ~75% of the genes in each genome were described in 25 *T. maritimum* strains [54]. In another study, 2034 core-genes were identified between 40 *T. maritimum* strains [55]. In this study, 3819.5 ± 2.1 CDS were similar between barcodes 9 and 10 using both 95% and 80% similarity thresholds. The large increase in the number of CDS compared and the amount of core-genes in this study, in contrast to previous work [54,55], could be attributed to factors including, but not limited to: the sample size and selected isolates (i.e., reduced sample size and geographically similar isolates for the same species would provide more similar core-genes); the sequencing platforms; how the genomes were assembled; the tools used to interpret core genes (Panaroo (this study) vs Microscope [54,55]); and potential contamination.

## 5. Conclusions

Ten *Tenacibaculum* isolates collected from mouthrot outbreaks in BC waters were sequenced with Oxford-Nanopore long-read sequencing technologies. Seven out of the ten isolates were assembled into circular and complete chromosomes. Larger genomic comparisons (i.e., ANI) provided improved species-level resolution in contrast to single gene comparisons. Average nucleotide identity analysis classified the isolates into four species (*T. maritimum* (T.mar 2.1C, T.mar ATR 174-1B) *T. finnmarkense* (20-4106-2, 17-2596-1, LI-C6 FM3-F), *T. dicentrarchi* (20–4116–9,18–3141), and *T. ovolyticum* (20-4135-2)), and two unknown novel species (*T. pacificus sp. nov*. type strain 18-2881-A^T^ and *T. retecalamus sp. nov*. type strain 18-3228-7B^T)^. Hemolysins were predicted in all of the barcodes, but several other putative toxins were predicted in *T. maritimum*. Few genes related to antimicrobial resistance were predicted, most notably genes related to oxytetracycline resistance. Subsequent work will focus on identifying whether the predicted genes inform virulence and antimicrobial resistance. This study is the first to describe the genomes of several *Tenacibaculum* species in Canada and BC waters, which will help inform future phylogenomic, virulence, and antimicrobial resistance research for *Tenacibaculum* spp. in BC.

## Figures and Tables

**Table 2:** National Center for Biotechnology Information (NCBI) sequences used in the FASTANI (v.1.33, https://github.com/ParBLiSS/FastANI, accessed on 28 November 2022, [39]) comparison.

Bacterial Sequence	Genbank Accession Number	NCBI Name
*Tenacibaculum adriaticum DSM 18961^T^*	GCF_008124875.1	ASM812487v1
*Tenacibaculum agarivorans HZ1^T^*	GCF_001936575.1	ASM193657v1
*Tenacibaculum aiptasiae a4^T^*	GCF_008806755.1	ASM880675v1
*Tenacibaculum caenipelagi CECT 8283^T^*	GCF_004363005.1	ASM436300v1
*Tenacibaculum dicentrarchi TD3509^T^*	GCF_900239455.1	TD3509TV1
*Tenacibaculum dicentrarchi TdCh04*	GCF_018616285.1	ASM1861628v1
*Tenacibaculum dicentrarchi TNO021*	GCF_900239305.1	TNO021V1
*Tenacibaculum dicentrarchi QCR29*	GCF_018616555.1	ASM1861655v1
*Tenacibaculum discolor DSM 18842^T^*	GCF_003664185.1	ASM366418v1
*Tenacibaculum finnmarkense gm. finnmarkense TNO006^T^*	GCF_900239185.1	TNO006V1
*Tenacibaculum finnmarkense gm. ulcerans TNO010^T^*	GCF_900239495.1	TNO010V1
*Tenacibaculum finnmarkense AY7486TD*	GCF_001483385.1	ASM148338v1
*Tenacibaculum finnmarkense TFHFJ ^T^*	GCF_900239485.1	TFHFJTV1
*Tenacibaculum gallaicum DSM 18841^T^*	GCF_003387615.1	ASM338761v1
*Tenacibaculum holothuriorum S2-2^T^*	GCF_002120225.1	ASM212022v1
*Tenacibaculum jejuense KCTC 22618^T^*	GCF_900198195.1	TjejuenseV1
*Tenacibaculum lutimaris DSM 16505^T^*	GCF_003610735.1	ASM361073v1
*Tenacibaculum maritimum NCIMB 2154^T^*	GCF_900119795.1	MARITPRJEB17743
*Tenacibaculum maritimum TM-KORJJ*	GCF_004803875.1	ASM480387v1
*Tenacibaculum mesophilum DSM 13764^T^*	GCF_003867075.1	ASM386707v1
*Tenacibaculum ovolyticum da5A-8*	GCF_001641405.1	ASM164140v1
*Tenacibaculum ovolyticum DSM 18103^T^*	GCF_000430545.1	ASM43054v1
*Tenacibaculum ovolyticum To-7Br*	GCF_021852385.1	ASM2185238v1
*Tenacibaculum pelagium S7007^T^*	GCF_014062345.1	ASM1406234v1
*Tenacibaculum piscium RT-G24*	GCF_021390715.1	ASM2139071v1
*Tenacibaculum piscium SC-I4*	GCF_021390755.1	ASM2139075v1
*Tenacibaculum piscium TNO020^T^*	GCF_900239505.1	TNO020V1
*Tenacibaculum piscium TNO070*	GCF_015143395.1	ASM1514339v1
*Tenacibaculum singaporense DSM 106434^T^*	GCF_003867015.1	ASM386701v1
*Tenacibaculum skagerrakense DSM 14836^T^*	GCF_004345825.1	ASM434582v1
*Tenacibaculum soleae UCD-KL19*	GCF_001693415.1	ASM169341v1
*Tenacibaculum todarodis LPB0136^T^*	GCF_001889045.1	ASM188904v1
*Tenacibaculum sp. SZ-18*	GCF_002813915.1	ASM281391v1
*Tenacibaculum sp. AHE14PA*	GCF_019278465.1	ASM1927846v1
*Tenacibaculum sp. AHE15PA*	GCF_019278445.1	ASM1927844v1

**Table 3:** *Tenacibaculum* isolate information, selection criteria, and presumed identity. Presumed identity was based on morphology ^1^, 16S rDNA qPCR, and 16S rDNA sequencing.

Isolate Name	Genomic Identification								
	qPCR (+/−) ^2^				16S (27F, 1492R) NCBI BLAST ^3^				Presumed Identity
	MAR	DICEN	FIN	OVO	Closest Match	Query Cover %, Similarity %, E-value			
20-4135-2	−	−	−	+	*Tenacibaculum ovolyticum* da5A-8	100	99.9	0	*T. ovolyticum*
20-4116-9	−	+	−	−	*Tenacibaculum dicentrarchi* TdChD04	99	98.8	0	*T. dicentrarchi*
20-4106-2	−	−	+	−	*Tenacibaculum finnmarkense* Tsp.2	100	99	0	*T. finnmarkense*
17-2576-1	−	+	+	−	*Tenacibaculum* sp. RTG-16	98	99.1	0	*T. finnmarkense*
18-2881-A	−	−	−	−	*Tenacibaculum* sp. Tsp.4	100	98.3	0	*Tenacibaculum sp.*
18-3228-7B	−	−	−	−	NA	NA	NA	NA	*Tenacibaculum sp.*
18-3141	−	+	−	−	NA	NA	NA	NA	*T. dicentrarchi*
LI C6 FM3-F	−	+	+	−	*Tenacibaculum finnmarkense* AY7486TD	100	99.9	0	*T. finnmarkense*
T.mar 2.1C	+	−	−	−	*Tenacibaculum maritimum* NLF-15	99	100	0	*T. maritimum*
T.mar ATR 174 1B	+	−	−	−	*Tenacibaculum maritimum* TFA4	99	98.9	0	*T. maritimum*

^1^ All isolates were yellow, Gram-negative, and elongated rod-shaped to filamentous. ^2^ For qPCR, a qualitative assessment occurred, ‘+’ indicates a positive reaction, ‘−’ indicates a negative reaction. DICEN and FIN [24], MAR [25], and OVO [26] assays were used. ^3^ All 16S rDNA BLAST comparisons used ~1300–1400 bp.

**Table 4:** *Tenacibaculum* barcode information describing the quality of data interpreted from basecalling, demultiplexing and other applied quality controls.

Barcode #	Isolate Name	Number of Reads	Total Basepairs	Estimated Chromosome Size	Estimated Chromosome Coverage	Read Length N50	Mean Read Length	Max Read Length	Mean Q Score
1	20-4135-2	46,497	345,317,009	4,100,000	84.22	13,519	7426.7	101,783	15.6
2	20-4116-9	112,128	732,515,487	2,700,000	271.3	9074	6532.9	81,239	15.2
3	20-4106-2	144,584	823,300,941	2,700,000	304.9	7801	5694.3	65,495	15.2
4	17-2576-1	216,705	994,359,562	2,700,000	368.3	6496	4588.5	63,050	15
5	18-2881-A	39,459	348,772,821	3,500,000	99.6	18,051	8838.9	117,965	15.3
6	18-3228-7B	57,597	460,320,495	3,500,000	131.5	15,516	7992.1	122,145	15.3
7	18-3141	108,120	763,100,153	2,700,000	282.6	9862	7057.9	64,381	15.3
8	LI C6 FM3-F	335,237	1233,306,653	2,700,000	456.8	4938	3678.9	66,955	15.2
9	T.mar 2.1C	154,709	663,990,855	3,300,000	201.2	6913	4291.9	119,184	15.3
10	T.mar ATR 174 1B	439,613	508,915,182	3,300,000	154.2	1255	1157.6	84,467	15.5

All barcodes were processed using ONT Guppy basecalling software (v.6.0.1+652ffd1) and the super high accuracy model (guppy/6.0.1/data/dna_r9.4.1_450bps_sup model). Modified demultiplexing included the command ‘trim_barcodes --min_score_barcode_front 70 -q 0 --detect_mid_strand_adapter --min_score_adapter_mid 65 --num_extra_bases_trim 80′. Reads shorter than 500 bp were omitted using filtlong (v 0.2.1).

**Table 5:** Genome assembly characteristics for barcodes 1–10.

Barcode #	Assembler ^1^ and Polishing Tool ^2^	Number of Identified Contigs	Complete Chromosomal Contig Size	Putative Plasmid Sizes *
1	Trycycler + Medaka	1	4.20 Mb	-
2	Raven + Medaka	5	1.6 Mb, 984 Kb	28 Kb, 14 Kb, 2 Kb
3	Trycycler + Medaka	2	2.86 Mb	22 Kb
4	Trycycler + Medaka	4	2.83 Mb	131 Kb; 14 Kb, 3 Kb
5	Trycycler + Medaka	1	2.79 Mb	-
6	Trycycler + Medaka	1	2.89 Mb	-
7	Trycycler + Medaka	1	2.78 Mb	-
8	Trycycler + Medaka	2	2.93 Mb	154 Kb
9	Raven + Medaka	5	1.9 Mb, 1.6 Mb, 700 Kb	5 Kb, 4 Kb
10	Raven + Medaka	45	Unclear	Unclear

^1^ Trycycler (v.0.5.1) or Raven (v.1.6.1); ^2^ Medaka (v.1.4.3); * inferred from circularization, agreement with Trycycler, and size [35].

**Table 6:** Average nucleotide identity comparison (%) summary comparing barcodes 1–10 against putative pathogenic *Tenacibaculum* species. Green highlighted cells represent *T. ovolyticum*, yellow indicates *T. dicentrarchi*, blue indicates *T. finnmarkense*, purple indicates *T. maritimum,* and grey indicates unclassified barcodes. Bolded cells represent over 95% nucleotide identity between intersecting query and reference sequences. All other comparisons can be found in Table S3.

ANI Comparison		Reference																			
		barcode1	barcode2	barcode3	barcode4	barcode5	barcode6	barcode7	barcode8	barcode9	barcode10	*T. maritimum* 2154^T^	*T. ovolyticum* DSM 18103^T^	*T. dicentrarchi* TD3509^T^	*T. finnmarkense gm. finnmarkense* TNO006^T^	*T. finnmarkense gm. ulcerans* TNO010^T^	*T. piscium* TNO020^T^	*T. soleae* UCD-KL19^T^	*T. mesophilum* DSM 13764^T^	*T. discolor* DSM 18842^T^	*T. gallaicum* DSM 18841^T^
**Query**	barcode1	100.0	81.4	81.1	81.2	82.5	83.4	81.3	81.2	78.4	78.3	78.3	97.6	81.5	81.1	81.3	80.6	83.5	78.3	79.6	79.3
	barcode2	81.6	100.0	94.1	94.1	88.3	82.0	99.2	94.1	79.2	79.7	78.1	81.5	98.2	94.1	94.1	87.3	81.7	79.0	79.1	79.0
	barcode3	81.2	94.1	100.0	98.6	87.5	81.9	93.9	96.9	78.4	78.0	78.0	81.0	93.4	98.2	96.9	87.7	81.4	78.5	78.8	78.6
	barcode4	81.2	93.9	98.6	100.0	87.2	81.9	93.9	96.9	78.1	78.6	77.8	80.9	93.3	98.2	96.8	87.4	81.3	78.5	78.7	78.7
	barcode5	82.6	88.3	87.4	87.2	100.0	83.1	88.1	87.3	78.1	78.3	78.0	82.4	87.9	87.2	87.3	85.6	82.2	79.4	79.5	79.3
	barcode6	83.6	82.1	81.9	81.8	82.8	100.0	82.0	81.7	78.1	78.0	78.1	83.5	81.8	81.8	81.6	81.3	85.3	79.8	80.0	79.7
	barcode7	81.5	99.2	94.1	94.0	88.0	82.0	100.0	94.1	78.4	78.1	78.2	81.5	98.1	94.1	94.2	87.1	81.6	79.0	79.0	79.1
	barcode8	81.3	94.0	96.9	96.9	87.2	82.0	93.8	100.0	78.0	78.6	77.9	81.1	93.5	96.8	98.6	87.9	81.2	78.7	78.8	78.9
	barcode9	78.2	79.0	78.1	78.3	78.0	78.0	77.9	78.1	100.0	99.9	97.4	78.0	78.2	78.0	78.2	78.4	78.1	78.0	78.0	77.8
	barcode10	78.3	80.0	78.7	79.3	78.4	78.2	78.8	79.0	99.9	100.0	97.4	78.1	79.0	78.7	79.0	78.9	78.2	78.1	77.9	77.8

**Table 7:** 16S rDNA investigation among annotated *Tenacibaculum* barcodes. Locus tags that were identical are within the same cells.

Barcode #	# Of Copies	# Of SNP	# Of INDEL	Length (bp)	Locus Tags	NCBI BLAST			
						Top BLAST Match	Query Cover	E-value	% Identity
1	7	1	0	1518	BIBBJE_07040 BIBBJE_14780	*T. ovolyticum* da5A-8 (LC144619.1)	100	0	99.93
					BIBBJE_08755 BIBBJE_14755 BIBBJE_15960 BIBBJE_15985 BIBBJE_17670	*T. ovolyticum* da5A-8 (LC144619.1)	100	0	100
2	9	28	1	1518	KGFFJA_05390	*T. finnmarkense* AY7486TD (CP013671.1)	100	0	98.88
					KGFFJA_05415	*T. finnmarkense* AY7486TD (CP013671.1)	100	0	98.75
					KGFFJA_05440 KGFFJA_10540	*T. dicentrarchi* 35/09^T^ (NR_108475.1)	99	0	98.94
					KGFFJA_05465	*T. dicentrarchi* 35/09^T^ (NR_108475.1)	99	0	98.91
					KGFFJA_07820	*T. dicentrarchi* 35/09^T^ (NR_108475.1)	99	0	98.28
					KGFFJA_09870	*T. finnmarkense* AY7486TD (CP013671.1)	100	0	98.75
					KGFFJA_12460	*T. finnmarkense* AY7486TD (CP013671.1)	100	0	98.88
				1517	KGFFJA_06620	*T. dicentrarchi* 35/09^T^ (NR_108475.1)	99	0	98.21
3	7	22	0	1518	DGBDCK_01985	*T. finnmarkense* AY7486TD (CP013671.1)	100	0	99.74
					DGBDCK_02660	*T. finnmarkense* AY7486TD (CP013671.1)	100	0	98.81
					DGBDCK_04865 DGBDCK_06085	*T. finnmarkense* AY7486TD (CP013671.1)	100	0	98.88
					DGBDCK_07235 DGBDCK_13085	*T. finnmarkense* AY7486TD (CP013671.1)	100	0	98.75
					DGBDCK_07260	*T. finnmarkense* AY7486TD (CP013671.1)	100	0	98.81
4	8	16	14	1520	CAJKLB_03075 CAJKLB_03965	*T. finnmarkense* AY7486TD (CP013671.1)	100	0	98.29
				1520	CAJKLB_07030	*T. finnmarkense* AY7486TD (CP013671.1)	100	0	98.35
				1522	CAJKLB_08670	*T. finnmarkense* AY7486TD (CP013671.1)	100	0	98.16
				1525	CAJKLB_10260	*T. finnmarkense* AY7486TD (CP013671.1)	100	0	98.36
				1525	CAJKLB_10285	*T. finnmarkense* AY7486TD (CP013671.1)	100	0	98.16
				1520	CAJKLB_10310	*T. finnmarkense* AY7486TD (CP013671.1)	100	0	98.22
				1522	CAJKLB_18445	*T. finnmarkense* AY7486TD (CP013671.1)	100	0	97.5
5	9	13	2	1518	JAJPGM_00905	*T. dicentrarchi* 35/09^T^ (NR_108475.1)	99	0	97.62
				1516	JAJPGM_01550	*T. dicentrarchi* 35/09^T^ (NR_108475.1)	99	0	97.28
				1518	JAJPGM_03165	*T. dicentrarchi* 35/09^T^ (NR_108475.1)	99	0	97.28
				1518	JAJPGM_04825	*T. dicentrarchi* 35/09^T^ (NR_108475.1)	99	0	97.62
				1518	JAJPGM_06265	*T. dicentrarchi* 35/09^T^ (NR_108475.1)	99	0	97.62
				1518	JAJPGM_06295	*T. dicentrarchi* 35/09^T^ (NR_108475.1)	99	0	97.68
				1518	JAJPGM_07025 JAJPGM_11610	*T. dicentrarchi* 35/09^T^ (NR_108475.1)	99	0	97.68
				1518	JAJPGM_07055	*T. dicentrarchi* 35/09^T^ (NR_108475.1)	99	0	97.68
6	7	14	2	1516	KPHGJK_03565	*Tenacibaculum* sp. AHE14PA (CP058983.1)	100	0	98.48
				1518	KPHGJK_07470	*Tenacibaculum* sp. AHE15PA (CP058982.1)	100	0	98.15
				1518	KPHGJK_07495	*Tenacibaculum* sp. AHE15PA (CP058982.1)	100	0	98.02
				1518	KPHGJK_07520	*Tenacibaculum* sp. AHE15PA (CP058982.1)	100	0	98.22
				1516	KPHGJK_07545	*Tenacibaculum* sp. AHE15PA (CP058982.1)	100	0	98.08
				1516	KPHGJK_13405	*Tenacibaculum* sp. AHE15PA (CP058982.1)	100	0	97.95
				1518	KPHGJK_13430	*Tenacibaculum* sp. AHE15PA (CP058982.1)	100	0	98.15
7	9	34	4	1516	APJPMD_02115	*T. finnmarkense* AY7486TD (CP013671.1)	100	0	98.81
				1518	APJPMD_02725	*T. dicentrarchi* 35/09^T^ (NR_108475.1)	99	0	99.07
				1518	APJPMD_04745	*T. finnmarkense* AY7486TD (CP013671.1)	100	0	98.88
				1518	APJPMD_05975	*T. dicentrarchi* 35/09^T^ (NR_108475.1)	99	0	99.27
				1518	APJPMD_07140 APJPMD_07165	*T. dicentrarchi* 35/09^T^ (NR_108475.1)	99	0	98.81
				1518	APJPMD_07190	*T. dicentrarchi* 35/09^T^ (NR_108475.1)	99	0	98.94
				1516	APJPMD_07215	*T. finnmarkense* AY7486TD (CP013671.1)	100	0	98.15
				1516	APJPMD_13045	*T. finnmarkense* AY7486TD (CP013671.1)	100	0	98.88
8	7	33	2	1518	FHDEPC_02035	*T. finnmarkense* AY7486TD (CP013671.1)	100	0	99.6
				1517	FHDEPC_02655	*T. finnmarkense* AY7486TD (CP013671.1)	100	0	99.67
				1518	FHDEPC_04750	*T. finnmarkense* AY7486TD (CP013671.1)	100	0	99.41
				1517	FHDEPC_06105	*T. finnmarkense* AY7486TD (CP013671.1)	100	0	99.34
				1517	FHDEPC_07265	*T. finnmarkense* AY7486TD (CP013671.1)	100	0	99.6
				1518	FHDEPC_07295	*T. finnmarkense* AY7486TD (CP013671.1)	100	0	99.67
				1517	FHDEPC_13480	*T. finnmarkense* AY7486TD (CP013671.1)	100	0	98.68
9	6	1	0	1520	KDPLPA_06585 KDPLPA_14230 KDPLPA_15870	*T. maritimum* TM-KORJJ (CP020822.1)	100	0	99.08
				1520	KDPLPA_14645 KDPLPA_17070 KDPLPA_18190	*T. maritimum* TM-KORJJ (CP020822.1)	100	0	99.14
10	8	NA	NA	793	FCGIAJ_04105 Partial	*T. maritimum* TM-KORJJ (CP020822.1)	100	0	96.2
				977	FCGIAJ_04905 Partial	*T. maritimum* TM-KORJJ (CP020822.1)	100	0	92.28
				1422	FCGIAJ_05975	*T. maritimum* TM-KORJJ (CP020822.1)	100	0	98.43
				1520	FCGIAJ_09820	*T. maritimum* TM-KORJJ (CP020822.1)	100	0	99.08
				1522	FCGIAJ_13200	*T. finnmarkense* AY7486TD (CP013671.1)	100	0	98.35
				1524	FCGIAJ_13230	*T. finnmarkense* AY7486TD (CP013671.1)	100	0	98.35
				1354	FCGIAJ_19415	*T. maritimum* TM-KORJJ (CP020822.1)	100	0	86.87
				1104	FCGIAJ_19615 Partial	*T. maritimum* TM-KORJJ (CP020822.1)	100	0	98.82

**Table 8:** Panaroo (v1.3.0) gene-cluster comparison among barcodes 1-10 at the 95% and 80% similarity threshold.

Group	Gene-Cluster Comparison			
	95%		80%	
	Total	Shared	Total	Shared
Genus (barcodes 1-10)	11,978	191	9960	973
*T. maritimum* (barcodes 9 & 10)	4215	3821	4196	3818
*T. dicentrarchi* (barcodes 2 & 7)	2591	2140	2626	2140
*T. finnmarkense* (barcodes 3, 4, & 8)	3197	2043	3265	2052
*T. dicentrarchi/T. finnmarkense (barcodes 2, 3, 4, 7,* & 8)	3655	1921	3724	1928

## Data Availability

The data is available through contacting the corresponding author. The assembled genomes are available on NCBI, under the BioSample accession numbers (SAMN32421886, SAMN32421887, SAMN32421888, SAMN32421889, SAMN32421890, SAMN32421891, SAMN32421892, SAMN32421893, SAMN32421894, SAMN32421895), and have also been annotated using NCBI’s Prokaryotic Genome Annotation Pipeline (PGAP) tool, post-publication.

## Supplementary Materials

The following supporting information can be downloaded at: https://www.mdpi.com/article/10.3390/pathogens12010101/s1, Figure S1: A MAUVE (v. 20150226 [Darling et al. 2004]) alignment of barcode 2 (A) and barcode 9 (B) to a reference genome. Barcode 2 is aligned to *T. dicentrarchi* AY7486TD (now *T. finnmarkense* [10]) and barcode 9 is aligned to *T. maritimum* NCIMB 2154^T^. The coloured areas represent similar matches between the barcode and the reference chromosome, the red boxes indicate the putative chromosomal contigs for barcode 2 and 9.; Figure S2: Individual, circular *Tenacibaculum* chromosome plots assembled from the Nanopore sequence data for barcode 1 (A) and 3–8 (B-G). Genomic elements are illustrated by Proksee (https://proksee.ca/, accessed on 28 November 2022). Orientation and origins are based on the rpiB gene. The characters displayed include coding DNA sequences (CDS), transfer-RNA (tRNA), ribosomal-RNA (rRNA), non-coding RNA (ncRNA), transfer-messenger RNA (tmRNA). The tracks outward in include the characters of the genome, guanine-cytosine (GC) content, GC skews (+/−), and rRNA.; Table S1: Nucleotide sequences of Bakta (v. 1.4.0 (https://github.com/oschwengers/bakta, accessed on 28 November 2022) annotated genes for barcodes 1-10.; Table S2: Locations of the loci in barcodes 1-10 used for phylogenomic comparisons.; Table S3: FASTANI (v. 1.33, https://github.com/ParBLiSS/FastANI, accessed on 28 November 2022 [Goris et al. 2007]) comparison of barcodes 1-10 (query) against barcodes 1-10 and NCBI sequences (reference). The output is provided as the average nucleotide identity (% ANI), number of orthologous matches, and total sequence fragments of query.; Table S4: FeGenie (v. 1.0, https://github.com/Arkadiy-Garber/FeGenie, accessed on 28 November 2022 [Garber, et al. 2020]) analysis of barcodes 1-10. The first sheet (i.e., Raw Output) described the output from FeGenie, describing the category of iron related protein, genome/assembly (i.e., barcode) used, the open reading frame (orf) the gene was found in, the matched protein based on hidden markov models (HMM), bitscore, bitscore cutoff, bitscore comparisons, and protein sequences. The second sheet (FeGenieSum.) is a summary of the first describing the number of the identified proteins within each category of iron related proteins for each barcode.; Table S5: Putative virulence factors described as toxins by [Pérez-Pascual et al. 2017] in barcodes 1–10.; Table S6: Comprehensive Antibiotic Resistance Database (CARD)-Resistance Gene Identifier (https://card.mcmaster.ca/analyze/rgi, (accessed on 14 November 2022)) output of barcodes 1-10 investigating antimicrobial resistance genes.; Table S7: Comparison of the genomic islands in barcodes 1–10 using IslandViewer4 (https://www.pathogenomics.sfu.ca/islandviewer, accessed on 28 November 2022, [Bertelli, et al. 2017]) to GYPSy v. 1.1.3 (Soares, et al. 2016). Plasmids were not compared. Both programs described the number of genomic islands, the loci within the genomic islands, and the number of tRNA, transposases, integrases, phage-related proteins, hypothetical proteins, and CDS within the genomic islands.
