# Supplementary figures and images for: Genomics of Tenacibaculum Species in British Columbia, Canada
Source: Pathogens. 2023 Jan 6;12(1):101. doi: 10.3390/pathogens12010101 (PMC9864904; doi:10.3390/pathogens12010101)

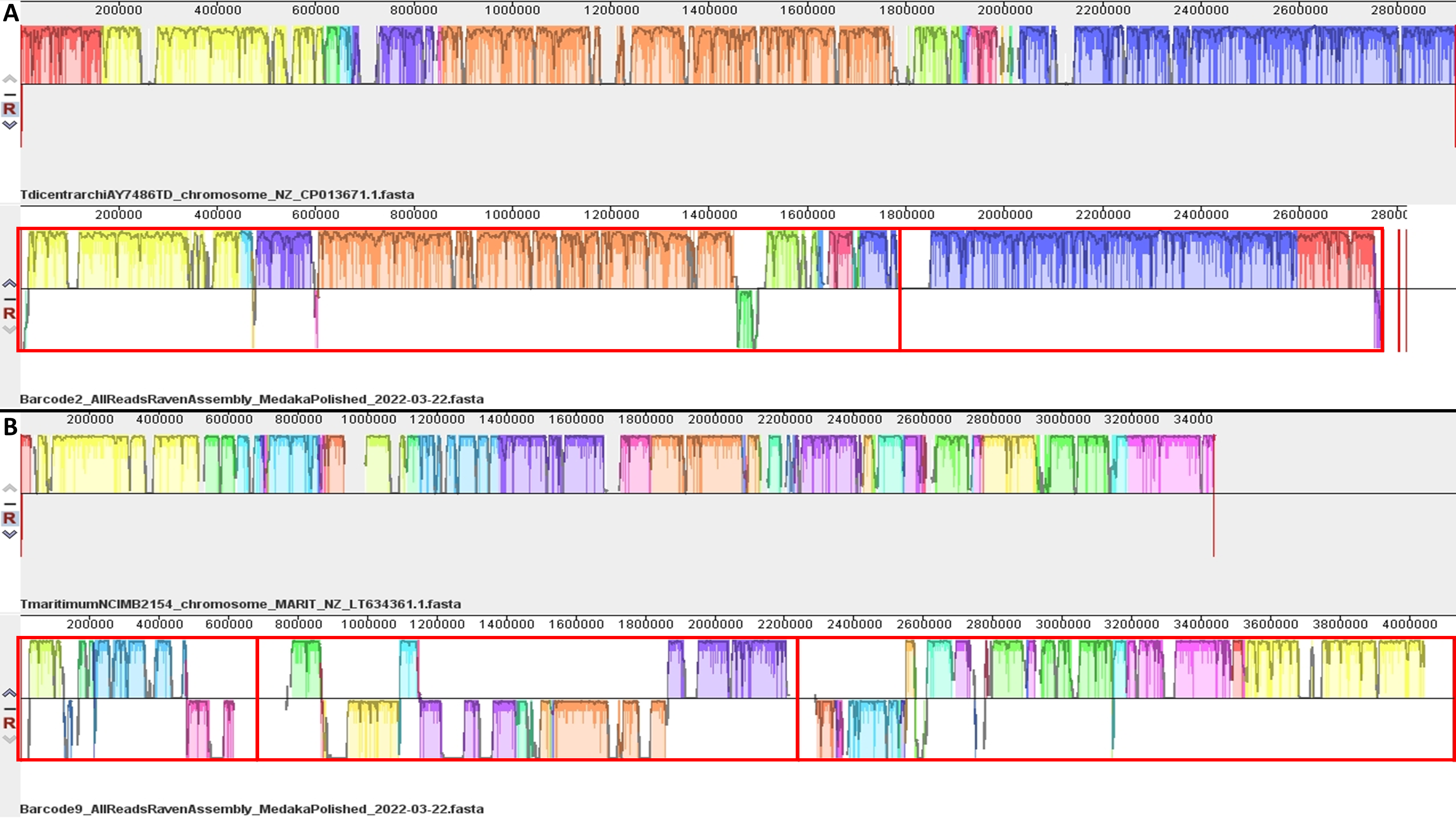

Supplement: Supplementary file 1 [file pathogens-12-00101-s001.zip › Supplementary Figures and Tables (Revised)/FigureS1_MAUVEalignmentsBC2_BC9_V2_09-11-2022.png]

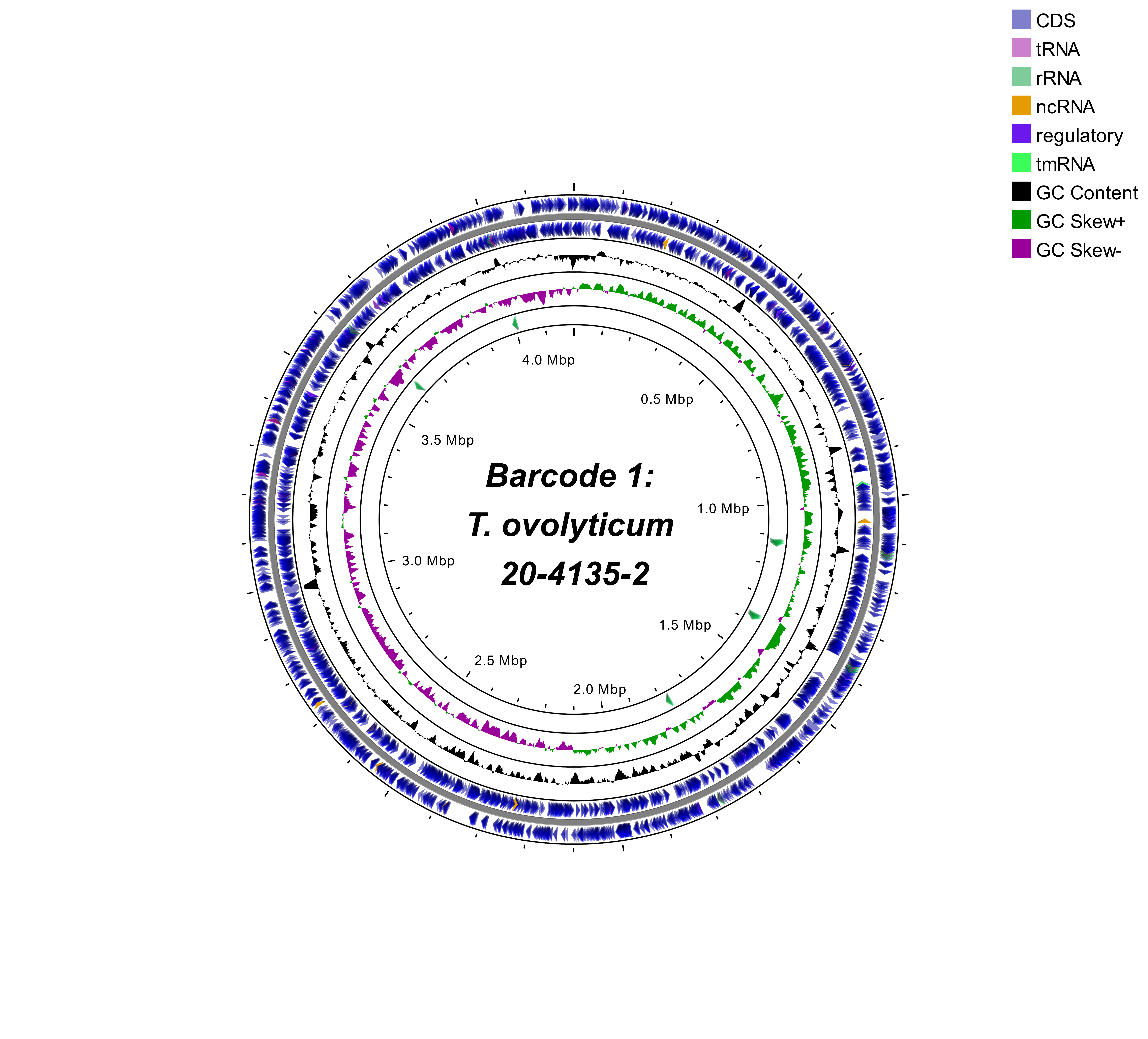

Supplement: Supplementary file 1 [file pathogens-12-00101-s001.zip › Supplementary Figures and Tables (Revised)/FigureS2_ProkseeGenomes/FigureS2-A-Barcode1.png]

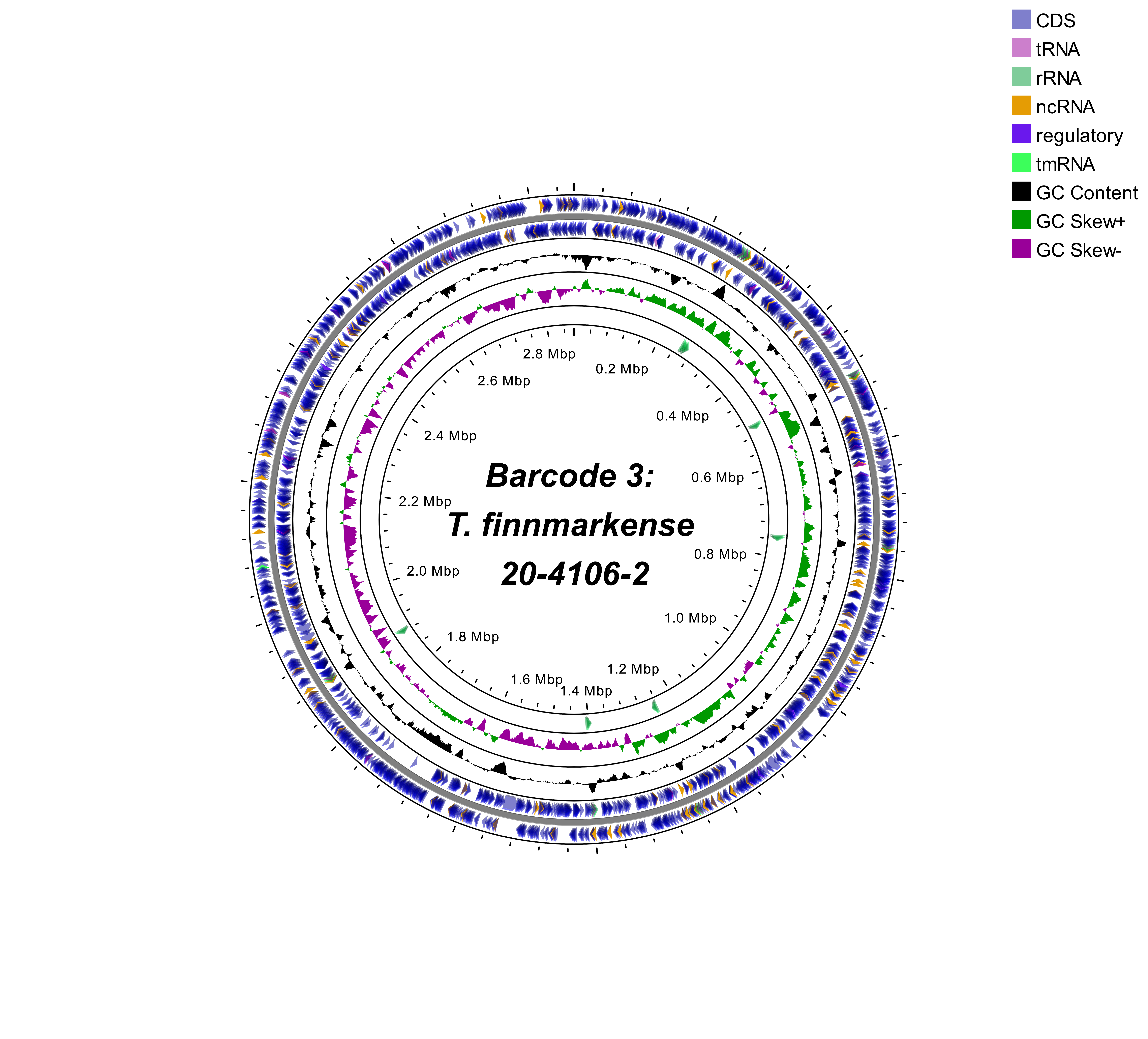

Supplement: Supplementary file 1 [file pathogens-12-00101-s001.zip › Supplementary Figures and Tables (Revised)/FigureS2_ProkseeGenomes/FigureS2-B-Barcode3.png]

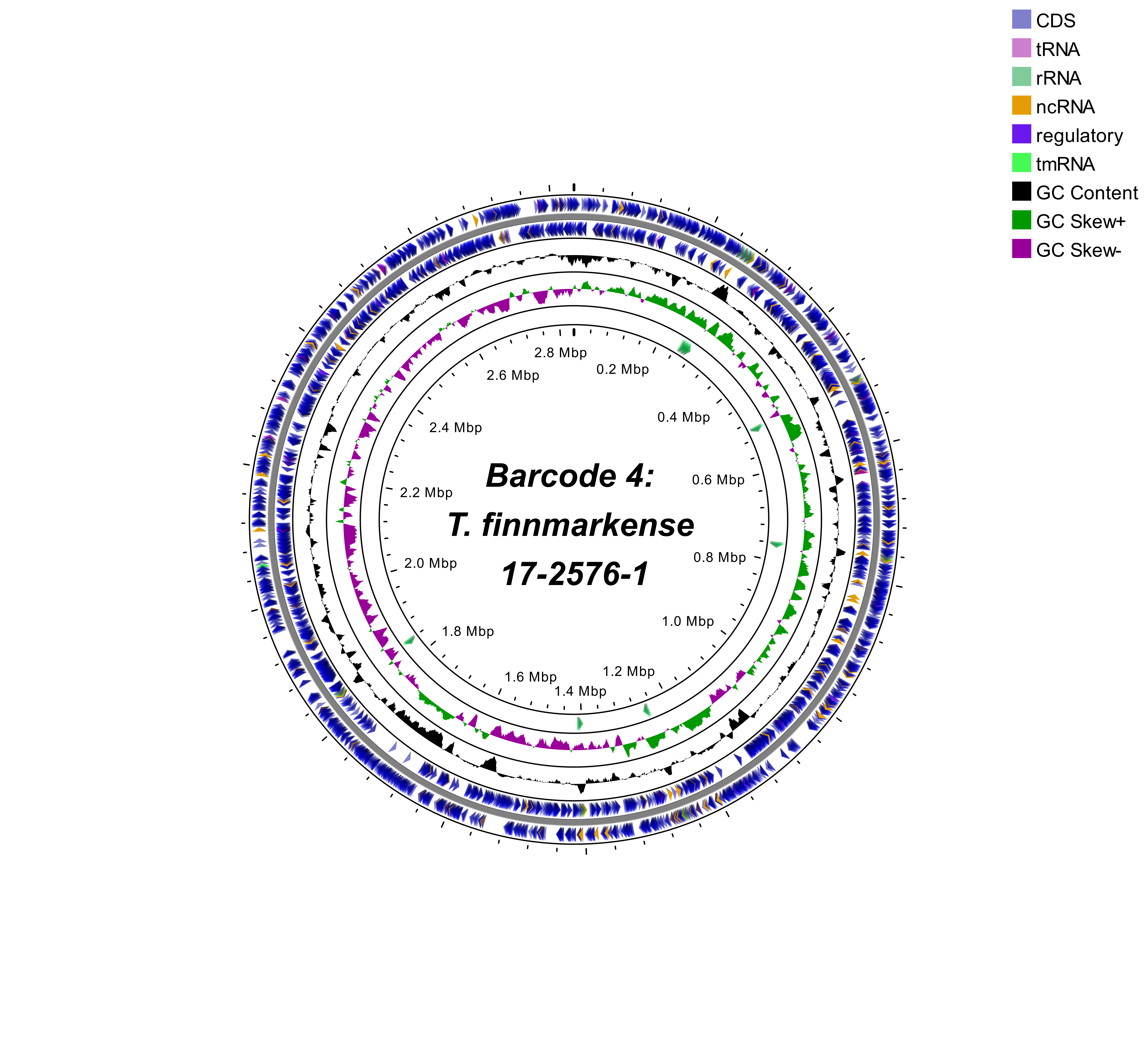

Supplement: Supplementary file 1 [file pathogens-12-00101-s001.zip › Supplementary Figures and Tables (Revised)/FigureS2_ProkseeGenomes/FigureS2-C-Barcode4.png]

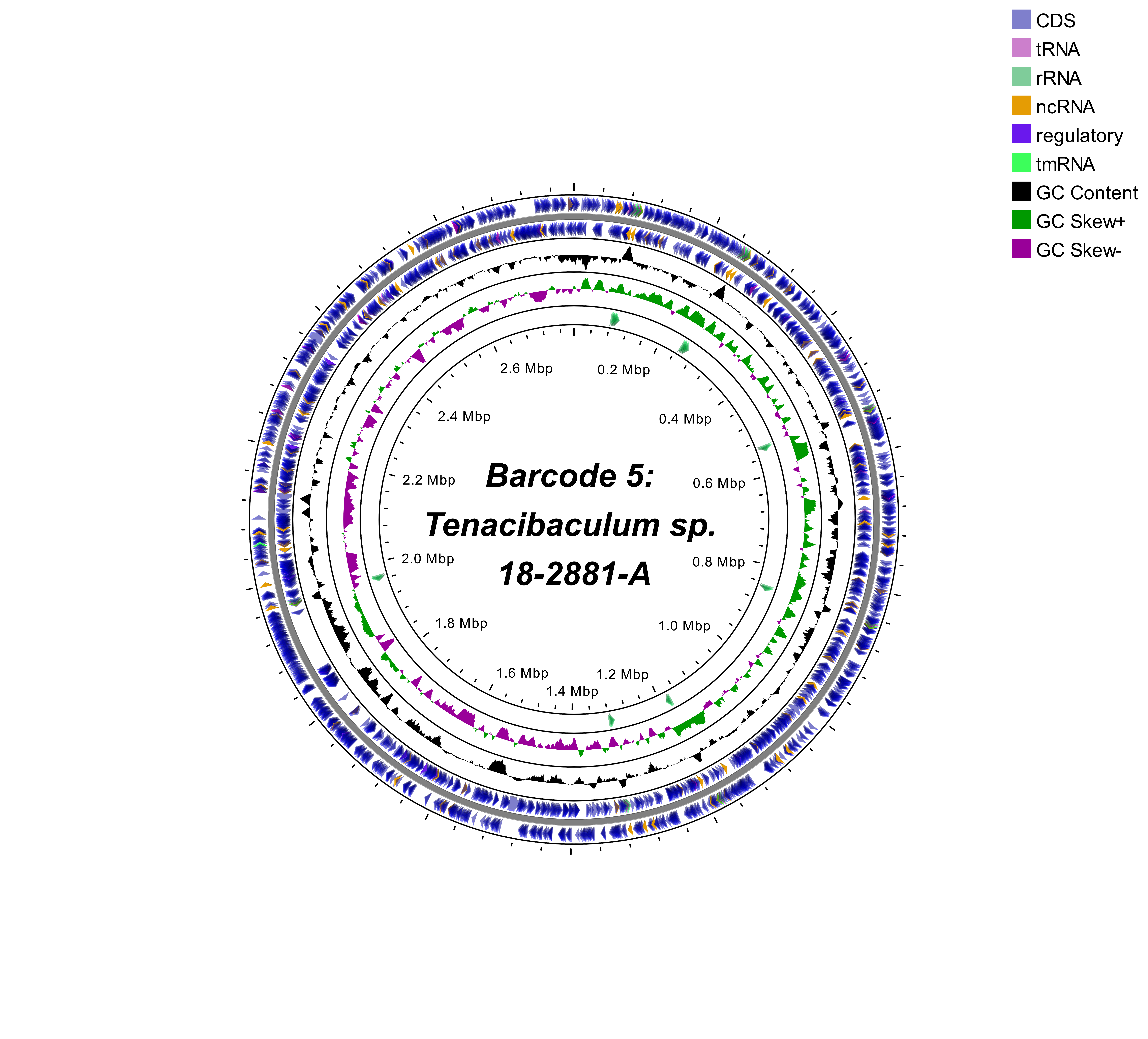

Supplement: Supplementary file 1 [file pathogens-12-00101-s001.zip › Supplementary Figures and Tables (Revised)/FigureS2_ProkseeGenomes/FigureS2-D-Barcode5.png]

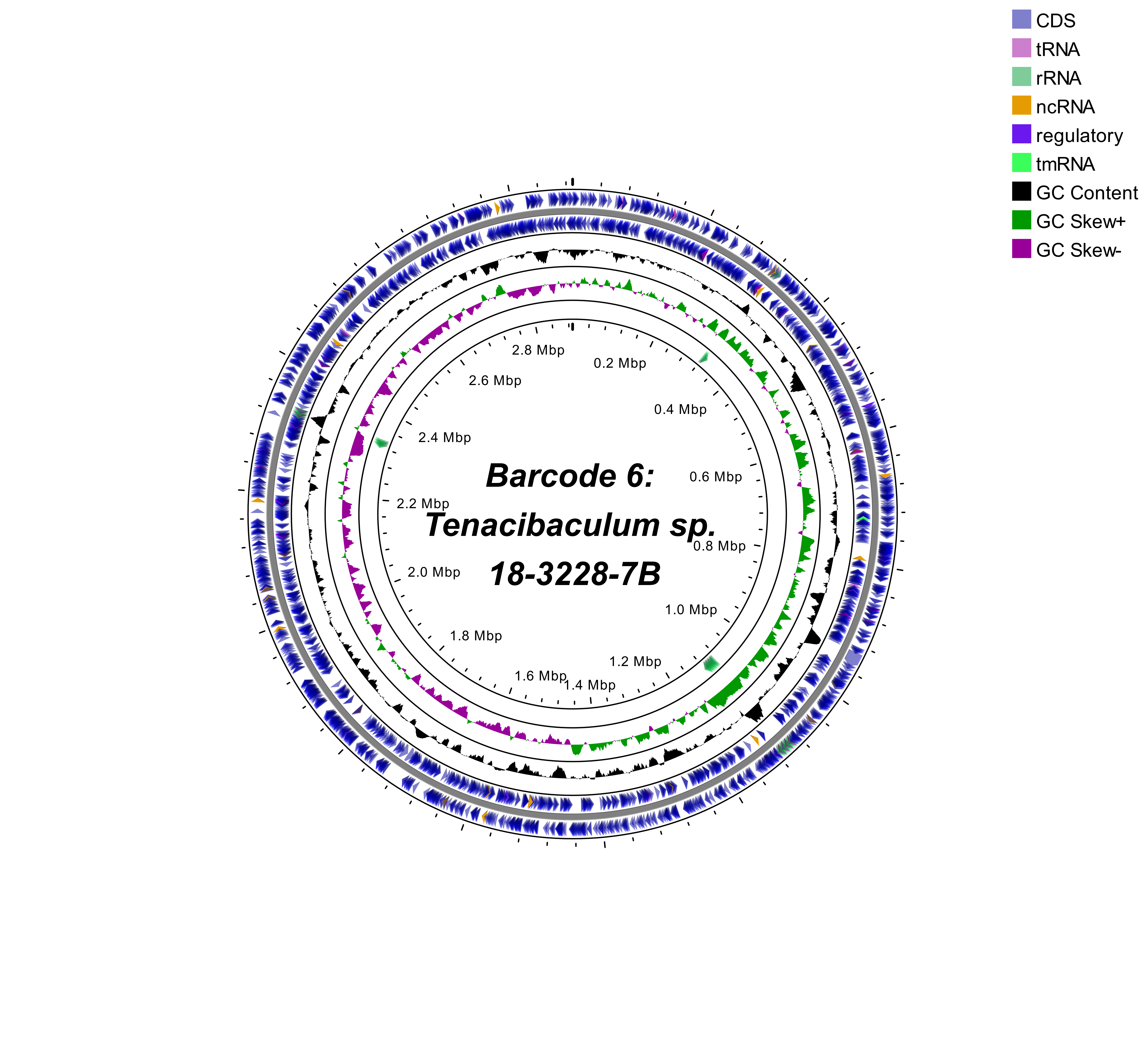

Supplement: Supplementary file 1 [file pathogens-12-00101-s001.zip › Supplementary Figures and Tables (Revised)/FigureS2_ProkseeGenomes/FigureS2-E-Barcode6.png]

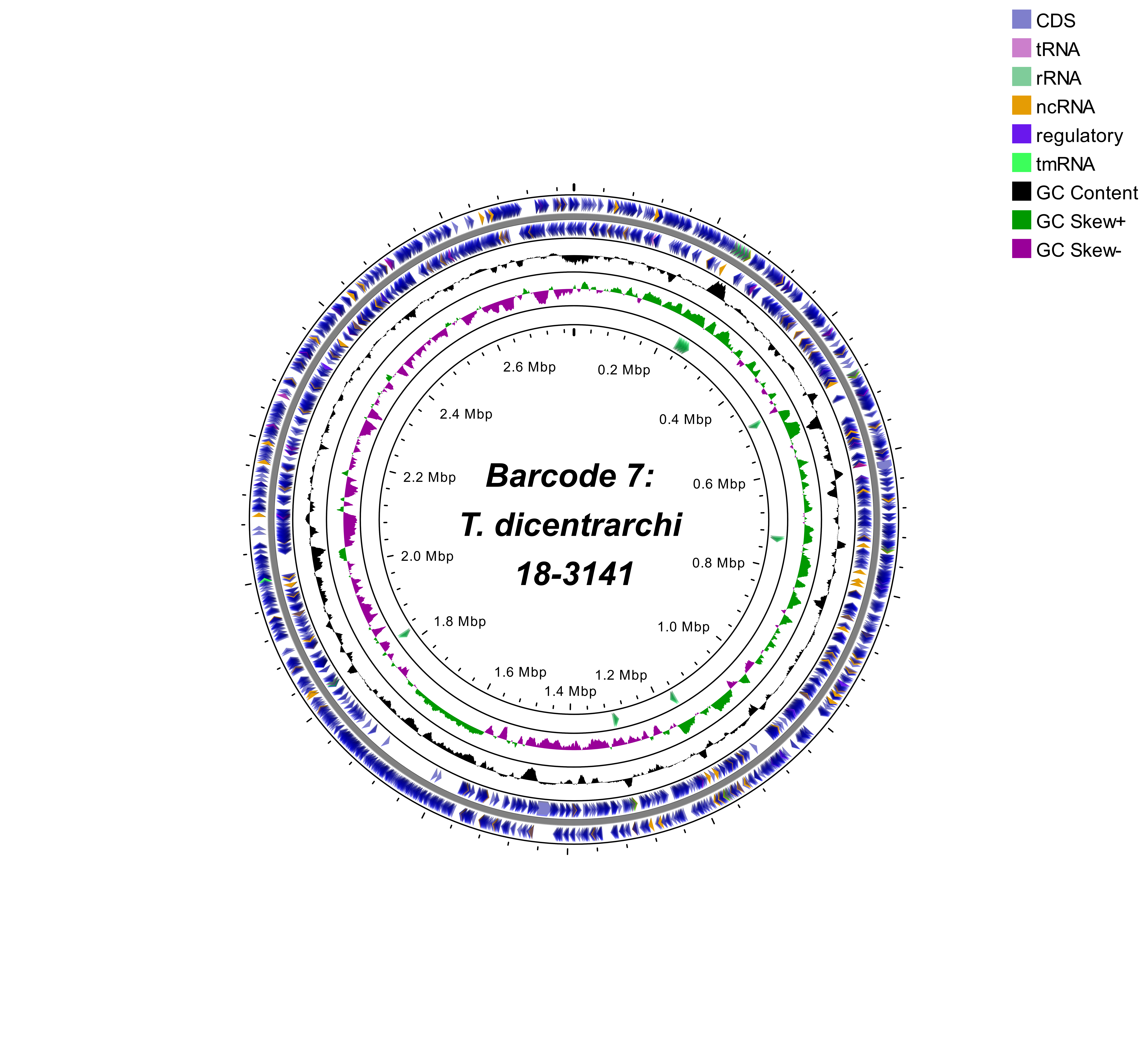

Supplement: Supplementary file 1 [file pathogens-12-00101-s001.zip › Supplementary Figures and Tables (Revised)/FigureS2_ProkseeGenomes/FigureS2-F-Barcode7.png]

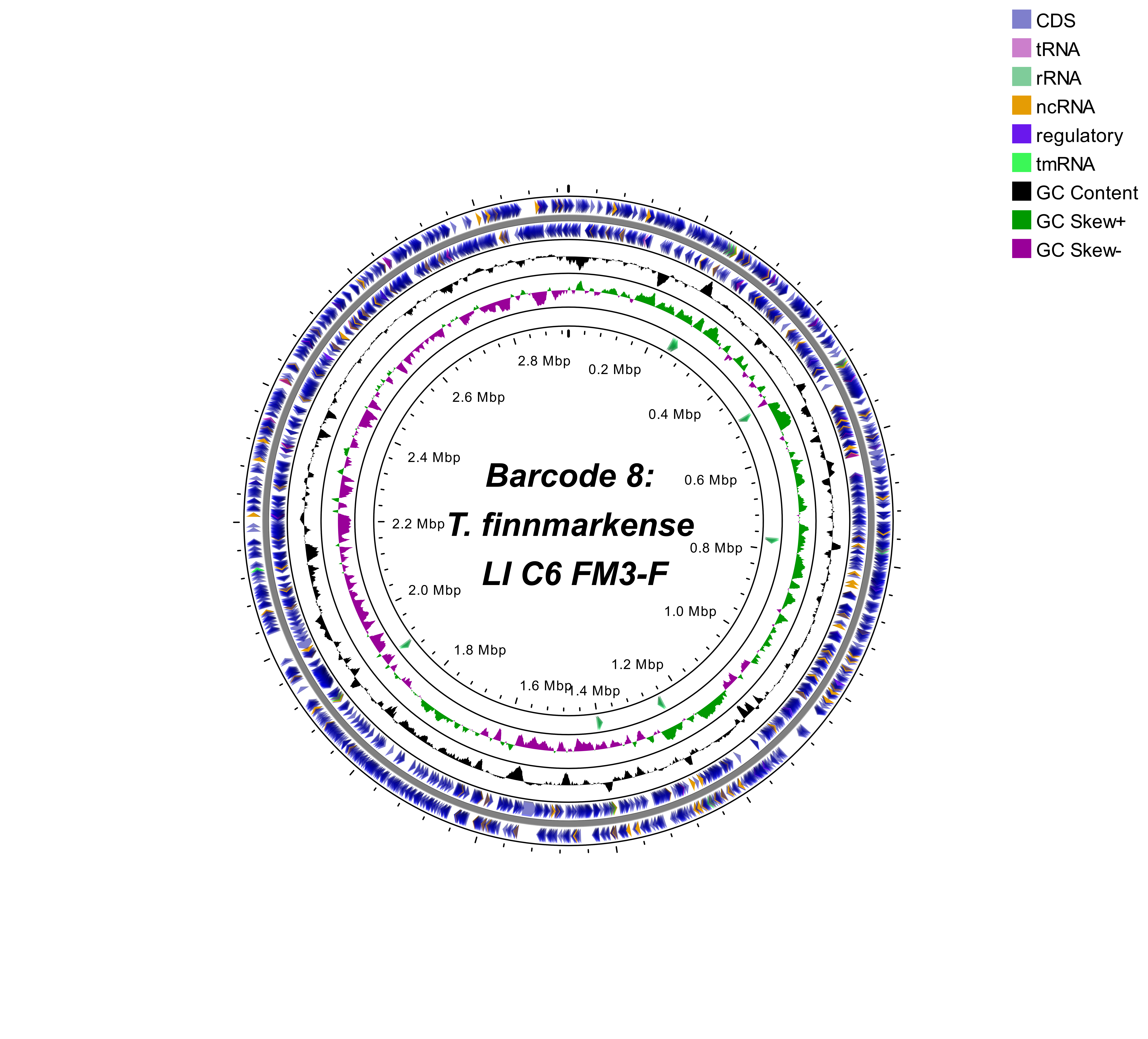

Supplement: Supplementary file 1 [file pathogens-12-00101-s001.zip › Supplementary Figures and Tables (Revised)/FigureS2_ProkseeGenomes/FigureS2-G-Barcode8.png]
